# Early etiology of Alzheimer’s disease: tipping the balance toward autophagy or endosomal dysfunction?

**DOI:** 10.1007/s00401-014-1379-7

**Published:** 2015-01-03

**Authors:** Aleksandar Peric, Wim Annaert

**Affiliations:** Laboratory for Membrane Trafficking, Department of Human Genetics, University of Leuven, and VIB-Center for the Biology of Disease, Gasthuisberg, O&N4, Rm. 7.159, Herestraat 49, 3000 Leuven, Belgium

**Keywords:** Alzheimer’s disease, Autophagy, Endolysosomal trafficking, Presenilins, γ-Secretase

## Abstract

Alzheimer’s disease (AD) is the most common form of dementia in the elderly. This brain neuropathology is characterized by a progressive synaptic dysfunction and neuronal loss, which lead to decline in memory and other cognitive functions. Histopathologically, AD manifests via synaptic abnormalities, neuronal degeneration as well as the deposition of extracellular amyloid plaques and intraneuronal neurofibrillary tangles. While the exact pathogenic contribution of these two AD hallmarks and their abundant constituents [aggregation-prone amyloid β (Aβ) peptide species and hyperphosphorylated tau protein, respectively] remain debated, a growing body of evidence suggests that their development may be paralleled or even preceded by the alterations/dysfunctions in the endolysosomal and the autophagic system. In AD-affected neurons, abnormalities in these cellular pathways are readily observed already at early stages of disease development, and even though many studies agree that defective lysosomal degradation may relate to or even underlie some of these deficits, specific upstream molecular defects are still deliberated. In this review we summarize various pathogenic events that may lead to these cellular abnormalities, in light of our current understanding of molecular mechanisms that govern AD progression. In addition, we also highlight the increasing evidence supporting mutual functional dependence of the endolysosomal trafficking and autophagy, in particular focusing on those molecules and processes which may be of significance to AD.

## Introduction

Alzheimer’s disease (AD) is a progressive neurodegenerative disorder and the most common form of dementia in the elderly. With the average life expectancy on the rise, AD is predicted to become a major socioeconomic burden in the near future. Extracellular amyloid plaques (APs) and intraneuronal neurofibrillary tangles (NFTs) are two major hallmark lesions of this fatal pathology [13]. Despite the significant advancement in our understanding of the mechanisms that contribute to AD progression, the effective disease-modifying/-ceasing drugs are still missing. In search for more optimal treatment avenues, AD researchers are increasingly considering combinatory approaches and shifting their focus to more fundamental disease-promoting events. To this end, alterations/dysfunctions in the endolysosomal–autophagic system are well-recognized early neuropathological features of AD, marked by prominent enlargement of endosomal compartments, progressive accumulation of autophagic vacuoles (AVs) and lysosomal deficits [102]. Lysosomes are major cellular degradative organelles, involved in turnover of molecular cargo from both autophagic and endocytic pathways, and in AD, disturbed lysosomal degradation is presumed to be of key importance in aberrant AV turnover. However, the principal causes of this dysfunction and the specific contribution of endosomal alterations herein are still debated, owing to the high complexity and the strong contextual dependence of AD pathogenesis. In this review, we summarize some of the findings pertaining to these issues in light of our current understanding of AD progression. We highlight the increasing evidence supporting the mutually dependent functioning of autophagy and endolysosomal trafficking regulators, particularly focusing on aspects of possible pathogenic importance in AD. Finally, we propose how insights from these early disease-promoting mechanisms could/should shape the development of novel therapeutic strategies toward the more efficient treatments for AD. The general modes of action and specific cellular functions of numerous autophagy and endolysosomal trafficking regulators are discussed only briefly, as they have already been summarized in other excellent reviews [48, 108], including some of this cluster (see e.g., Damme et al. [26]). We limit our focus to molecules/processes relevant to AD.

## Etiology of Alzheimer’s disease

AD pathology is associated with a progressive deterioration of memory and other cognitive functions. In most prevalent sporadic cases, the disease has a late onset. Here, the time between the initiation of cognitive decline and death is highly variable, ranging from years to over a decade. The main risk factor in AD is age; however, even though one in three people older than 85 years will become affected by this pathology, the disease itself is not a simple outcome of aging. For instance, rare familial AD (FAD) forms have an early onset and many additional genetic and environmental risk factors influence the more common sporadic AD (SAD) pathology [13]. Symptomatic manifestations of AD reflect impaired functioning of specific brain areas, where underlying pathogenic processes result in a progressive dysfunction/degeneration of synapses/neurites and eventual loss of vulnerable neurons [13]. Assuming that APs and NFTs are disease-causing alterations, the majority of the research efforts in the AD field initially focused on these lesions, revealing that amyloid beta (Aβ) peptide aggregates and hyperphosphorylated tau fibrils, respectively, are their prominent constituents [13]. Despite our growing understanding of this disease, how exactly these AD hallmarks relate to specific pathogenic processes, however, still remains enigmatic.

## Aβ: mechanisms of generation and toxicity

**Fig. 1:**
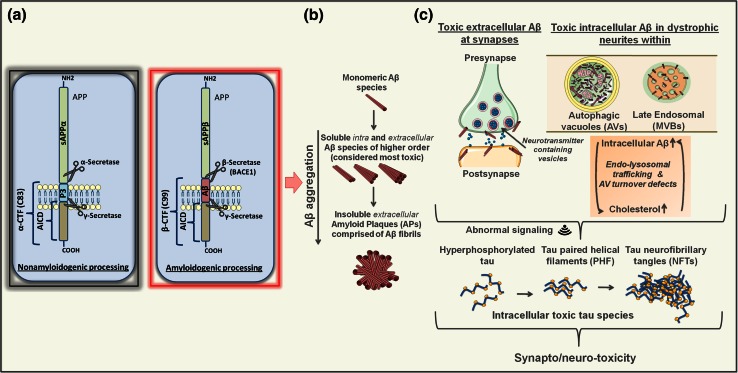
Amyloidogenic vs. nonamyloidogenic APP processing, Aβ aggregation and intracellular vs. extracellular Aβ toxicity. **a** Non-amyloidogenic processing of APP (*left*) requires the dual proteolysis, first by members of the ADAM (a disintegrin and metalloprotease domain-containing) protein family (mainly, ADAM10 and ADAM17, also called α-secretases) followed by γ-secretase, resulting in release of soluble sAPPα ectodomain, a non-amyloidogenic p3 fragment and APP intracellular domain (AICD). In the amyloidogenic pathway (*right*), APP is first cleaved by β-secretase BACE1 releasing the sAPPβ ectodomain, followed by γ-secretase processing of the remaining β-CTF giving rise to AICD and Aβ peptide species of slightly different lengths. **b** Monomeric Aβ species, particularly Aβ42 and Aβ43, have a tendency to aggregate and form structures of higher order. These include toxic soluble Aβ dimers, trimers, oligomers and protofibrils, found both inside and outside of the cells/neurons, as well as more inert insoluble amyloid fibrils, which comprise extracellular APs. **c** In AD, in addition to their direct effects on synaptic transmission/integrity via binding to synaptic membranes/receptors, toxic Aβ species may also accumulate within dystrophic neurites and aberrant synaptic regions in intracellular compartments, including late endosomal multivesicular bodies (MVB) and autophagic vacuoles (AVs) [144, 172]. Indicated as well is the hypothesized self-propelling exacerbating influence of excessive Aβ/cholesterol accumulation, disturbed endolysosomal trafficking regulation and/or defective turnover of autophagic vacuoles (AV) in this context. All together, this could lead to aberrant cellular signaling that in turn may induce and propagate excessive tau phosphorylation, accumulation of toxic tau species and related synapto/neurotoxic effects

Aβ peptides are produced by a consecutive cleavage of amyloid precursor protein (APP) by beta-site APP-cleaving enzyme 1 (BACE1, or β-secretase), and γ-secretase, a transmembrane protein complex, which in humans consists of anterior pharynx-defective 1 (APH-1A/1B), presenilin enhancer 2 (PEN-2), nicastrin (NCT) and catalytically active presenilin 1 or 2 (PSEN1/2) [28, 138]. This dual amyloidogenic scission of APP, which in cells/neurons competes with the non-amyloidogenic processing mechanisms, yields several peptide species of slightly different length, among which the 40 amino acid residues long (Aβ40) is the most abundant form [28]. Longer Aβ42/43 species, however, have a higher propensity to aggregate and are therefore considered more neurotoxic [28, 123] (Fig. 1a, b). They are believed to be a main driver of neurodegeneration in AD, as many FAD-associated dominant mutations in the genes encoding APP and PSENs increase their total and/or relative amounts compared to Aβ40 [22, 28]. While this provides a strong support for the disease-promoting role of Aβ in context of certain early-onset FAD cases, the precise contribution of these peptide entities to SAD is still elusive. Also, the primary sites of Aβ toxic activity and the contribution of its aggregation status herein continue to be debated.

### Extracellular vs. intracellular Aβ and its aggregation in the pathogenesis of AD

The presumed pathogenicity of extracellular Aβ is based on the fact that these aggregation-prone peptides are secreted into the external environment where plaques are found [133]. In this case, amyloidogenic processing of APP would result in Aβ liberation and its consequent spontaneous self-aggregation into amyloid forms of higher order, including Aβ fibrils, which precipitate in APs. Insoluble APs, however, correlate poorly with neuronal loss [45] and dementia [5], and nowadays their Aβ oligomeric precursors are considered to be more neurotoxic [160]. In AD, these soluble Aβ forms associate better with disease severity [88] and dementia [87], and display stronger correlation with synaptic loss [79]. Here, their excessive binding to synaptic membranes/receptors is believed to underlie consequent cognitive deficits [94]; however, whether all aspects of Aβ toxicity are exclusively mediated via their pathogenic influences from the extracellular environment remains an intriguing question (Fig. 1b, c).

To this end, in recent years, there is a growing awareness that the intraneuronal pool of Aβ as well may be detrimental in AD. In an APP-based murine AD model, in which enhanced oligomerization of Aβ occurs without its fibrillization, it is noted that the time of initial deterioration of synaptic function and memory coincides with intraneuronal Aβ accumulation [150]. Also, in 3xTg-AD and arcAβ mice, accumulation of intraneuronal Aβ correlates with early cognitive deficits, preceding the appearance of APs [11, 65]. In APP(SL)/PS1KI mice, in turn, internal pile up of Aβ, rather than its external deposition in APs, associates with neuronal loss [23]. In primary neurons and brains of Tg2576-AD APP mutant mice as well as in human AD brains, intracellular Aβ42 accumulates and oligomerizes within late endosomal multivesicular bodies (MVBs) of neuronal processes and synaptic compartments, where its deposition associates with their morphological abnormalization [144]. In line, in both AD and Down syndrome patients (that invariably develop features of AD neuropathology due to trisomy of chromosome 21 and thus an extra *APP* copy) early rises in Aβ coincide with its deposition within abnormally enlarged neuronal endosomes [19]. Dystrophic neurites and synaptic terminals in AD also prominently accumulate AVs [101], which not only can facilitate the Aβ clearance [62], but also may be an important source of amyloidogenic activity [172]. Together, this implies that part of the pathogenic mechanisms in AD could also affect the homeostasis of the cellular interior, where internalized and/or internally produced Aβ may be a factor of synapto/neurotoxicity (Fig. 1c).

### Endosomal trafficking/sorting regulation in amyloidogenesis

Indeed, increasing evidence suggests that in neurons/cells amyloidogenic processing of APP preferentially occurs internally, e.g., within the biochemically optimal endosomal compartments, where specific protein trafficking/sorting regulators can impact the Aβ production [113, 125]. In this respect, in AD context, the role of retromer and its accessory proteins is becoming prominent. This heteropentameric adaptor protein complex, comprising two subcomplexes (the VPS35/26/29 trimer and hetero/homodimer of sorting nexins (SNXs)), facilitates protein cargo recognition and transport from endosomes to the *trans* Golgi network (TGN) or plasma membrane [131]. While this is primarily important in the maintenance of an active pool of lysosomal hydrolase receptors in TGN, retromer-mediated sorting also controls intracellular shuttling of other proteins, including Aβ yielding APP and BACE1 [113, 162, 163]. Notably, in AD-vulnerable entorhinal cortex, the levels of both VPS35 and VPS26 are specifically decreased [135]. The importance of this AD-related change is underscored by findings demonstrating that hemizygous deletion of *VPS35* in Tg2576 mice elevates the hippocampal Aβ levels and exacerbates the AD pathology [162]. There is also strong evidence that APP sorting receptor SORLA/LR11/SORL1 (sortilin-related receptor), genetically linked to AD, is as well decreased and/or dysfunctional in this disorder [163]. SORLA controls the intracellular APP trafficking by associating with the retromer and other sorting adaptors [163]. Like in the case of VPS35, its deficiency has been implicated in enhanced Aβ production and AD pathogenesis [34]. Although there is still no clear consensus with respect to how exactly retromer-mediated intracellular sorting of APP and BACE1 affects the Aβ production, a currently prevailing idea postulates that retromer dysfunction may disrupt normal trafficking of APP and BACE1, thereby increasing the likelihood of their physical co-sequestration in endosomal vesicles, and thus promote amyloidogenesis (for a review see [113, 163]). In line, other retromer-associated sorting receptors genetically linked to increased AD risk, like, e.g., SORCS1, may play a similar role [68]. Interestingly, in addition to APP, SORLA via its N-terminal VPS10P domain may also directly bind Aβ and thus regulate its trafficking to lysosomes for degradation. This function is disturbed by an FAD mutation in *SORL1*, which compromises its interaction with Aβ [17]. Endosomal sorting defects related to decreased levels of phosphatidylinositol-3-phosphate (PI3P), required for proper functioning of retromer and other sorting regulators, were recently also associated with AD and shown to underlie aberrant amyloidogenic processing [93]. Finally, intracellular sequestration of cholesterol, a relevant risk factor in AD [13], can as well promote abnormal endosomal trafficking/activity of amyloidogenic proteins, thus enhancing Aβ production [81, 119, 167].

### Toxic effects of Aβ on endolysosomal–autophagic system

In addition to being produced within intracellular compartments, Aβ can also disturb their normal functioning. To this end, recent genome-wide genetic screen in yeast, accompanied by congruent findings in nematode glutamatergic and rat primary cortical neurons [151], identified several endocytic regulators as modifiers of Aβ42 toxicity. This among others included the ortholog of human PICALM (phosphatidylinositol binding clathrin assembly protein), a regulator of clathrin-mediated endocytic trafficking and one of the most well-established SAD risk factors [151]. The importance of endosomally localized Aβ indeed cannot be underestimated, because a failure in either degrading or secreting it may increase its local concentration within these acidic compartments, thus facilitating its oligomerization and subsequent pathogenic processes [41, 51, 144]. Accordingly, AD-associated APP “Arctic” mutation (APP_arc_; E693G) favors the formation of soluble Aβ protofibrils as well as the intracellular amyloidogenic APP processing [99, 122]. Pile up of oligomeric Aβ42 in endosomes in turn may hamper cholesterol efflux from these compartments [91], thus creating a vicious cycle of self-propelling endosomal dysfunction and excessive Aβ production (see above and Fig. 1c). Localized to endosomes, Aβ42 may also affect their sorting capacity, thereby causing degradation and signaling defects [1]. Importantly, excessive levels of oligomerized Aβ42 may even compromise the physical integrity (impermeability) of endolysosomal–autophagic compartments [31, 41, 74, 144, 155, 169]. For instance, an in vivo overexpression of Aβ42 in fruit fly (*Drosophila melanogaster*) neurons causes progressive impairment of their degradative capacity and buildup of increasingly dysfunctional AVs [74]. Here, at early stages, AVs are protective and contribute to Aβ42 elimination, but as toxic burden increases, impaired degradation and leakage of lysosomal proteins from abnormal AVs promote neurodegeneration [74]. Taken together, intracellular Aβ accumulation may be both a cause and a consequence of an endolysosomal–autophagic dysfunction in AD (Figs. 1c, 4).

## Molecular origins of NFTs: tau phosphorylation and its pathological functions in AD

NFTs are insoluble intraneuronal fibrillary aggregates comprised of hyperphosphorylated microtubule-binding protein tau, which are readily observed in relation to AD and several other neurodegenerative tauopathies [8]. Tau protein is primarily found in neurons of the central nervous system, where it mainly localizes to axons and to a lesser extent neuronal soma and dendrites [12]. Here, tau stabilizes neuronal microtubules and regulates axonal transport of molecular cargo between the cell body and the distant synapses [32]. In neurons, tau, however, may also act as a specialized protein scaffold, thereby taking part in various signal transduction cascades [47]. Phosphorylation is one of the major posttranslational modifications of tau, which contributes to fine-tuning of its physiological functions both in development and adulthood [161]. In neurodegenerative pathologies, including AD, abnormal tau phosphorylation, however, disrupts its normal functioning, resulting in its self-aggregation into paired helical filaments that form NFTs [2, 8] (Fig. 1c). Notably, while the anatomically defined blueprint of NFT spreading is a well-established correlate of dementia and AD severity [16], increasing data suggest that here prefilamentous tau oligomers may in fact be a more relevant contributing factor to early toxicity [95].

Although tau plays an important role in AD pathogenesis, no FAD mutations have been found in its *MAPT* gene, arguing against an initial causality for developing AD. Familial mutations in tau, however, do exist in a subset of frontotemporal lobar degeneration (FTLD) pathologies, called frontotemporal dementia with parkinsonism linked to chromosome 17 (FTDP-17). Here, filamentous intraneuronal inclusions of hyperphosphorylated tau found in carriers of these mutations strongly reinforce the importance of this protein and its excessive phosphorylation in neurodegeneration in general [18, 165].

Importantly, in AD context, Aβ-mediated neurotoxicity seems to require tau [55, 117]. Accordingly, Aβ is able to modulate tau phosphorylation and the extent of NFT burden [46, 149]. Recent data also show that Aβ-associated clinical decline in cognitively normal older individuals occurs only in relation to elevated phospho-tau [30]. Therefore, in the pathogenic cascade of events leading to tau hyperphosphorylation, Aβ likely acts as an upstream modulator.

How abnormal tau phosphorylation contributes to AD pathology is nevertheless still enigmatic. On one hand, tau deficiency is largely protective against Aβ toxicity, suggesting that in AD tau may gain a toxic function [55, 117]. However, in certain contexts, lack of tau may as well be detrimental, as shown by crossing Tg2576-AD mice with tau^−/−^ animals [27]. While this suggests that loss of function mechanisms cannot be excluded as potential pathogenic modality for tau, increasing evidence implies that toxic gain of function by this protein may after all be more relevant. One of the current hypotheses postulates that in AD, tau-related toxicity may result from its mislocalization to neuronal soma/dendrites and/or excessive phosphorylation that would render aberrant interactions with molecules with which it either would not normally interact or would do so to a lesser extent [47]. Notably, these tau pathogenic activities may affect processes in both axonal and dendritic compartments. For instance, tau phosphorylation-dependent retention of the kinesin complex component, c-Jun N-terminal kinase-interacting protein 1 (JIP1) in neuronal soma, provides a possible explanation for impaired axonal transport in AD [56]. In turn, in the context of dendritic roles of tau, its interaction with the Src kinase Fyn was shown to be pivotal in Aβ-mediated excitotoxicity via a mechanism involving tau-dependent shuttling of Fyn to postsynaptic sites [55]. As phosphorylation of tau can promote its interaction with Fyn [10] as well as its postsynaptic targeting and consequent early synaptic deficits [50], abnormal localization, phosphorylation and interactions of this protein in dendrites may all be relevant AD promoters (Fig. 1c). While the precise spatio-temporal relationship in this cascade of pathogenic events in AD is slowly emerging, the key mechanisms responsible for the aberrant tau phosphorylation are still obscure. Based on certain pathomechanistic analogies between AD and Niemann–Picks disease type-C (NPC), we hypothesize that here deficits in endolysosomal–autophagic system may, at least in part, underlie the abnormal activity of enzymes controlling the extent of tau phosphorylation.

### Endolysosomal–autophagic dysfunction and tau phosphorylation: lessons from NPC

NPC is an autosomal recessive lysosomal storage disorder, caused by mutations in *NPC1* or *NPC2* genes and characterized by late endosomal/lysosomal accumulation of several lipid species, including unesterified cholesterol [156]. Interestingly, this fatal neurodegenerative disease displays some intriguing parallels to AD with respect to certain aspects of cellular pathology. In addition to similar exacerbating influence of cholesterol and intracellular Aβ42 deposition in endosomes [58], this also includes pronounced endolysosomal–autophagic abnormalities [36, 58, 73] as well as aberrant tau phosphorylation [78, 128]. Because neither in AD nor NPC tau is mutated, its abnormal phosphorylation in the context of these diseases therefore likely results from deregulated levels/activity of tau kinases and/or phosphatases. To this end, it is noteworthy that many of these enzymes (reviewed in [82, 83] and summarized in Table 1) take part in various cellular signaling cascades, in which endosomal membranes play an important regulatory function as signaling platforms [90, 108]. Because a similar role in cellular signaling control has recently also been ascribed to certain autophagy regulators and autophagosomal membranes [84, 85], it seems plausible to assume that anomalies in endolysosomal–autophagic system, common to both NPC and AD, may at least in part explain the aberrant activity of enzymes regulating tau phosphorylation. Notably, abnormal functioning of the endolysosomal–autophagic system, in addition, may contribute to pile up of toxic tau species by hampering their clearance (as their turnover relies on autophagy and lysosomal function) [109]. Accordingly, autophagy deficits have recently been directly implicated in tau phosphorylation and related neurodegeneration [52]. Moreover, intracellular accumulation of oligomeric Aβ, known to promote endolysosomal–autophagic defects (see above), coincides with early rises in abnormal tau phosphorylation in an APP-based AD model with endogenous (unmutated) tau, long before APs are formed [150]. Overall, this strongly supports the pathogenic relevance of disturbed intracellular trafficking/degradative homeostasis in AD in general and tau pathology in particular (Fig. 1c). Additional evidence reinforcing this concept comes from studies demonstrating that AD-related and γ-secretase-associated PSENs, may independent of their proteolytic function affect the endolysosomal–autophagic system as well as tau phosphorylation.

**Table 1:** Protein kinases and phosphatases implicated in regulation of tau phosphorylation

	Full name
GSK3	Glycogen synthase kinase-3
CDK5	Cyclin-dependent protein kinase-5
Erk1/2	Extracellular signal-regulated kinase 1/2
JNK1–3	c-Jun N-terminal kinase 1–3
P38	P38 kinase
CK1/2	Casein kinase 1/2
PKA	Protein kinase A
CaMKII	Calcium- and calmodulin-dependent protein kinase-II
TTBK1/2	Tau tubulin kinase 1/2
PKC	Protein kinase C
PhK	Phosphorylase kinase
PKB (Akt) 1–3	Protein kinase B 1–3
DYRK1A/2	Dual-specificity tyrosine phosphorylation and regulated kinase-1A/2
PKN	Protein kinase N
MARK 1–4	Microtubule affinity-regulating kinase 1–4
SFK	Src family kinases (Src, Lck, Syk, Fyn)
c-Abl	c-Abelson kinase
(Arg) kinase	Abl-related gene kinase
PP-2A	Protein phosphatase 2A
PP-1	Protein phosphatase 1
PP-2B (PP3)	Protein phosphatase 2B (calcineurin)
PP5	Protein phosphatase 5
PTEN	Phosphatase and tensin homolog deleted on chromosome 10

Note that the above listed enzymes can affect the extent of tau phosphorylation directly and/or indirectly, by regulating the activity and/or the ability of other kinases/phosphatases to phosphorylate/dephosphorylate tau at specific serine, threonine or tyrosine residues (as reviewed in [82, 83])

### γ-Secretase-independent function of PSENs in the endolysosomal–autophagic system

**Fig. 2:**
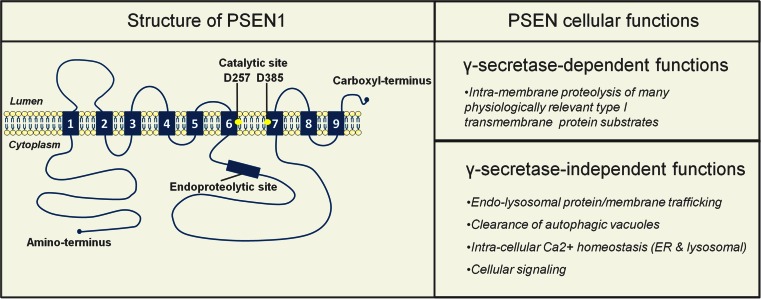
γ-secretase-dependent and -independent functions of presenilins (PSENs). PSEN1 (and likely PSEN2) has nine transmembrane domains (TMDs) [139], with two aspartate residues (D257/D385; *yellow circles*) in TMD 6 and 7 forming the catalytic core [138]. Full-length PSEN1 is endoproteolyzed in early secretory compartments resulting in stable PSEN1-NTF and -CTF heterodimers [138]. The catalytic role of PSENs, as part of the γ-secretase complex, is associated with the processing of around 100 currently known substrates [60]. PSENs, however, also have γ-secretase-independent functions, including roles in endolysosomal protein/membrane trafficking and clearance of autophagic vacuoles [37, 69, 116, 164], intracellular Ca^2+^ homeostasis (ER [98, 153] and lysosomal [24, 96]) and cellular signaling [6, 7, 33, 116]

PSENs are primarily known to be catalytic components of the γ-secretase complex that—besides APP—cleaves many other type I transmembrane proteins, thus taking part in a wide range of cellular processes [28, 60]. However, other functions, distinct from their role in intramembrane proteolysis have been attributed to the PSENs as well, including cellular signaling, intracellular Ca^2+^ homeostasis, endolysosomal trafficking and autophagy (Fig. 2). Particularly interesting seems that, in both in vitro and in vivo settings, using various cell types, primary neuronal cultures as well as murine brain samples, PSEN deficiency was shown to result in endolysosomal–autophagic abnormalities [33, 37, 69, 97, 164]. For instance, in adult murine brain neurons, with both PSEN isoforms genetically ablated, such defects occur very early (already at 2–3 months after birth) [69]. Around the same time these mice start having synaptic and memory deficits, which worsen with age due to progressive neurodegenerative alterations, accompanied by aberrant tau phosphorylation [127], implying an important role of PSENs in all these phenomena. Additional evidence indicates that much like lack of PSENs, also certain of their FAD-linked mutations can alter the intracellular signaling, leading to pathological tau phosphorylation [7] and in vitro degeneration of primary neurons [6], in a manner independent of their catalytic activity [6, 7]. FAD-related PSEN mutations also associate with pronounced lysosomal neuropathology in AD neurons [20], which based on evidence from FAD patient fibroblasts may compromise their degradative function [69]. In line, other studies demonstrate that some of the PSEN1 FAD mutants, unlike wild-type human PSEN1 (hPSEN1) and its catalytically inactive forms, are unable to fully rescue altered Wnt signaling in PSEN-deficient cells [33] or completely alleviate defective epidermal growth factor receptor (EGFR) turnover in lysosomes, in a similar context [116]. Together, all these studies suggest that FAD-associated PSEN mutations, in addition to altering the catalytic activity of this protein, may also contribute to disease progression via additional loss of other functions. As PSEN-dependent endolysosomal–autophagic and signaling phenotypes are even more pronounced when PSENs are lacking [33, 69, 116], their overall decreased levels, per se, may also be important. In support, polymorphisms are found in the *PSEN1* promotor sequence that repress transcription of PSEN1 and associate with both increased risk for AD and elevated total Aβ load [146]. Progressive lowering of PSEN1 expression in vitro, paralleled by concomitant gradual increase in Aβ42 levels (observed in another study) [115], accordingly reinforces the assumption that such mechanisms are possible and potentially relevant, also in amyloidogenesis. To this end, structural (conformational) changes in PSEN1, similar to those observed in some of its FAD mutants, were recently shown to occur in relation to SAD and aging, wherein they were proposed to underlie pathogenic amyloidogenesis [159]. Based on this, it seems tempting to speculate that in AD, altered levels, structure and activity of PSEN proteins may all be relevantly important and that their catalytic function may work together with γ-secretase-independent roles in disease promotion.

**Fig. 3:**
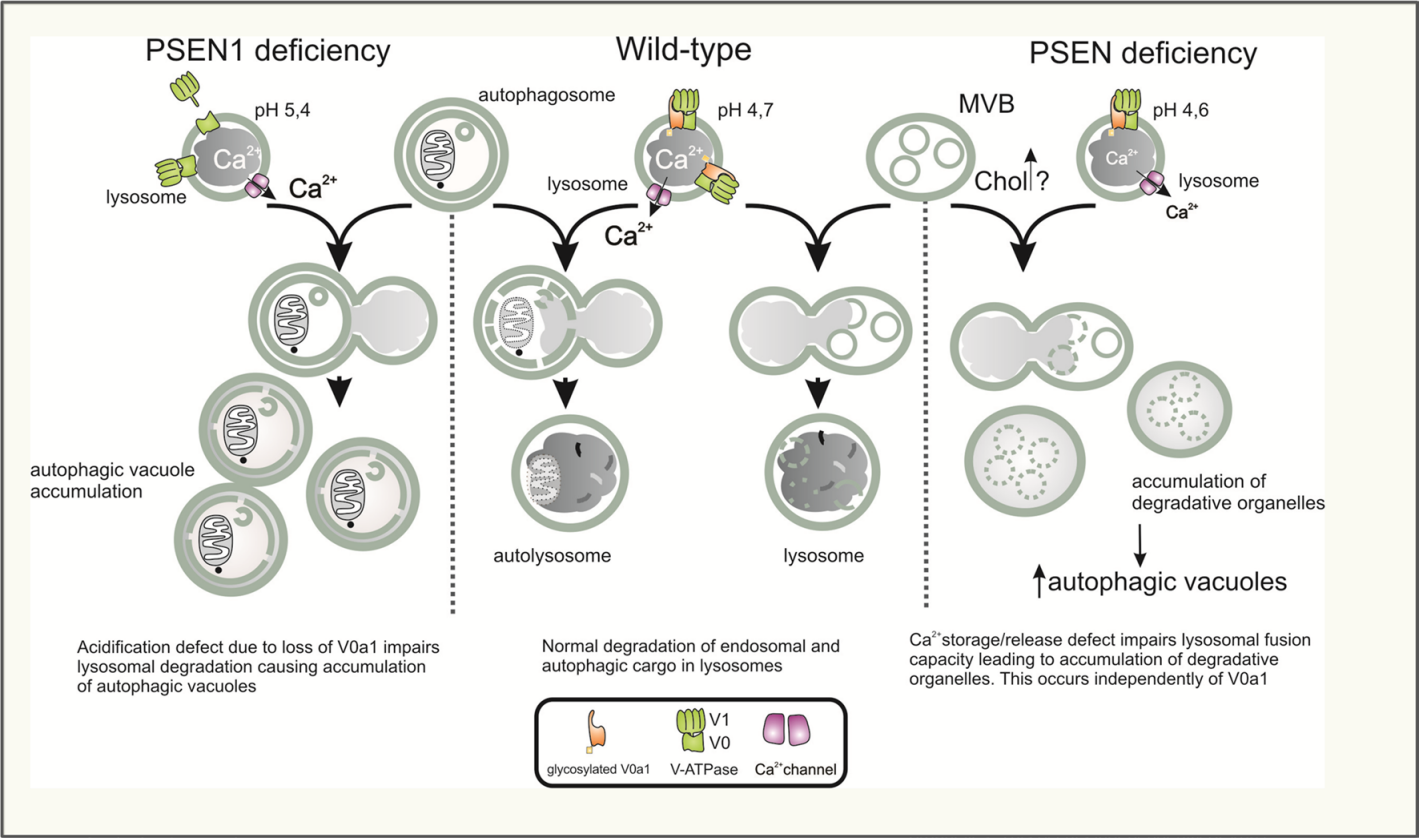
Presenilins (PSENs) and lysosomal degradation. Impaired lysosomal degradation observed in PSEN-deficient cells is attributed to either a failure in lysosomal acidification (*left*) or disturbed lysosomal calcium release/storage (*right*). *Left* a defect in lysosomal acidification is here primarily caused by the failure of the V0a1 subunit of the V-ATPase (proton pump) to become N-glycosylated, resulting in a dysfunctional proton pump, higher pH and decreased lysosomal degradative capacity. This in turn is claimed to underlie the accumulation of autophagic vacuoles (AVs) [69]. Alternatively (*right*), a deficit in lysosomal calcium storage/release affects the fusion of degradation-prone vesicles (late endosomes (MVBs) and AVs) with lysosomes [24, 37], thus compromising their clearance. Here, PSEN-dependent lysosomal Ca^2+^ defects could relate to alterations in endosomal trafficking homeostasis (endosomal recycling and normal endosomal maturation), which may lead to a buildup of lipids like cholesterol (Chol) and/or mislocalization of relevant transporters and channels, all of which could underlie the observed deficits (see the main text for clarifications). The middle panel depicts undisturbed fusion/degradation with/in lysosomes in cells with normal levels of PSENs

One of the first clear indications of a γ-secretase-independent role of PSEN1 in endolysosomal–autophagic system emerged from the identification of its interaction with ICAM-5 (also named telencephalin) [3]. This forebrain-specific neuronal intercellular cell adhesion molecule with an exclusive somatodendritic localization is, despite its type I transmembrane topology, not a γ-secretase substrate [37]. Instead, in PSEN1^−/−^ primary hippocampal neurons, ICAM-5 accumulates intracellularly in degradative organelles that are not acidified, but label positively for certain autophagic markers [37]. While these accumulations also occur in wild-type (WT) neurons, PSEN1 deficiency clearly leads to their earlier and more abundant appearance [37, 111]. Accumulation of similar degradative organelles was also noted in another study, where PSEN1 deficiency in neurons was shown to lead to a pronounced α- and β-synuclein intracellular accumulation [164]. Later, Lee and co-workers supported these seminal observations by identifying extensive accumulations of autophagic compartments in PSEN1-deficient cells, as well as in the brains of PSEN1 hypomorph or conditional knockout mice [69]. Importantly, in addition to extensive accumulation of AVs, PSEN-deficient cells were also shown to have significantly more late endosomal MVBs [33]. Although all studies agree that the observed phenomena relate to an impaired turnover of endosomal/autophagic cargo, whether lysosomal degradation per se or their fusion capacity is compromised, remains debated. To explain these deficits, two major hypotheses have been put forth, including defective lysosomal acidification and Ca^2+^ homeostasis (Fig. 3).

Lee et al. suggested a model whereby PSEN1 deficiency/FAD-related mutations would compromise a proclaimed role of endoplasmic reticulum (ER)-localized full-length PSEN1 as a critical co-factor of the oligosaccharyltransferase (OST) complex in N-glycosylation of the V0a1 subunit of the vacuolar ATPase (v-ATPase; proton pump). Allegedly, this should hamper the targeting of V0a1 to lysosomes, thus compromising the proton pump function and consequently lysosomal acidification and degradation (by impairing the activity of lysosomal hydrolases) [69]. In contrast, we as well as others failed to reproduce pronounced acidification defects [24, 97, 175], related to it disruption of lysosomal proteolysis and cathepsin D maturation [24, 175] and, critically important for the original hypothesis, defective N-glycosylation of the V0a1 subunit in PSEN-deficient cells [24, 175]. Using *Drosophila* *melanogaster* as a model, we conclusively showed that embryonic lethality caused by the lack of the V0a1 ortholog v100 could be fully rescued by a glycosylation-deficient v100 mutant, underscoring that N-glycosylation is even dispensable for the proper lysosomal targeting and function of this proton pump subunit [24]. Overall, this raises doubt if defective lysosomal acidification and the proposed mechanism, in particular, indeed primarily underlie PSEN-related lysosomal deficits. Alternatively, we originally demonstrated that lysosomal calcium storage/release, which is as well required for lysosomal function and fusion, is compromised in PSEN-deficient cells and neurons [24]; a finding later confirmed by others [96] (Fig. 3).

This phenomenon resembles the situation in NPC cells where a significant reduction in lysosomal calcium storage/release irrespective of acidification defects causes similar endolysosomal dysfunction. The initiating factor here is an aberrant sphingosine storage that instigates altered calcium homeostasis leading to secondary sphingomyelin and cholesterol storage [76]. In line, NPC disease-associated endolysosomal dysfunction can be induced by decreasing and rescued by increasing the Ca^2+^ levels [76]. Rescue of Ca^2+^ defects in PSEN-deficient cells in turn can be achieved by catalytically inactive hPSEN1 [24], underscoring a γ-secretase-independent nature of PSEN-mediated lysosomal Ca^2+^ regulation.

Toward identifying the underlying causes of this lysosomal dysfunction, our recent omics study performed on isolated plasma membranes (PMs) of PSEN-deficient cells already provide first insights. Using a novel isolation procedure, based on superparamagnetic nanoparticles, we compared the biomolecular composition of pure PMs derived from PSEN double knockout (PSENdKO) mouse embryonic fibroblasts (MEFs) vs. their WT and hPSEN1 rescued counterparts [147]. Our experiments revealed a convergent prominent surface depletion of cholesterol along with certain proteins, of which many are constituents of focal adhesion sites, lipid rafts and caveolae (or functionally related to them), in PSEN deficient cells [147]. Several of these molecules are also small GTPases involved in endosomal transport regulation [147]. As these PSEN-dependent surface alterations go along with a decrease in focal adhesion sites and caveolae, intracellular accumulation of cholesterol, caveolin-1 and the integrin-interacting CD47 protein [147, 166], we hypothesize that PSENs may regulate selective intracellular trafficking routes. Among the surface-depleted GTPases in PSENdKO MEFs, several play a role in endosomal recycling, such as RAB10, RAB11 and RAB35. RAB11 is a known interactor of PSENs [35], also recently shown to be of potential relevance to AD and amyloidogenesis [154]. RAB35, on the other hand, has mutually antagonizing roles with ADP-ribosylation factor 6 (ARF6) in endocytosis and endosomal sorting/recycling, important in regulation of cellular adhesion, migration, phagocytosis, cytokinesis and neurite outgrowth [21]. Interestingly, ARF6 has recently been found to be functionally linked as well to both APP processing and PSEN1 interactors. First, we showed that ARF6 plays a role in surface to endosome transport of BACE1 via the clathrin-independent internalization route, thereby keeping this amyloidogenic sheddase spatially separated from APP (the internalization of which is primarily clathrin dependent) until they meet in early endosomes [126]. Here, affecting ARF6 activity or expression inversely impacted on APP processing and Aβ production, thereby underscoring the important role of sorting/recycling regulation mediated by ARF6 in amyloidogenesis [126]. Second, and related to PSEN1, ARF6 also regulates the surface expression and later endosomal routing of the PSEN1 interactor ICAM-5 (which accumulates intracellularly in PSEN1^−/−^ neurons; see above) [37, 111]. While the exact role of ARF6 in these PSEN-related trafficking defects remains to be elucidated, it seems interesting that NPC-associated aberrant cholesterol efflux from endosomes can be induced by blocking and rescued by promoting ARF6-mediated recycling [130]. Aberrant cholesterol efflux in NPC also affects amyloidogenic APP processing by trapping APP and BACE-1 in the same endosomal compartments [81], thus further extending parallels to ARF6-mediated transport regulation in amyloidogenesis. Based on these studies it seems tempting to speculate that a defect in selective protein/membrane routing (to recycling and/or degradation) may contribute to or even underlie the endolysosomal–autophagic dysfunction observed in relation to PSEN deficiency and/or different PSEN FAD mutants (Fig. 3). As similar deficits are also noted in SAD, analogous pathogenic mechanisms, disturbing specific trafficking/degradative processes, may also operate in late-onset AD. Note, however, that depending on the context (FAD vs. SAD) the primary pathogenic factors may differ. To provide further support for this concept, in the following paragraphs we will highlight the known functional links between the autophagy and the endolysosomal trafficking regulators, which may be of potential pathogenic significance in AD.

## (Macro)autophagy, a major neuroprotective stress response pathway: functional links with endolysosomal trafficking regulators and their potential roles in AD

Aging is still the primary risk factor in AD [13]. Macroautophagy, (hereafter called autophagy) in turn, is one of the main quality control systems in cellular homeostasis and a major regulator of longevity [157], triggered by various stressors, including nutrient scarcity, hypoxia, oxidative stress, infection as well as the accumulation of aggregated (aggregate-prone proteins) and dysfunctional organelles [66]. Autophagy proceeds in a stepwise manner, via the initiation, elongation, maturation and degradation phases, during which its cytoplasmic targets are first engulfed by autophagosomal membranes to eventually become delivered to lysosomes for degradation (see Damme et al. [26] from this cluster of reviews for an updated overview of molecular mechanisms of autophagy progression and selective cargo clearance).

**Fig. 4:**
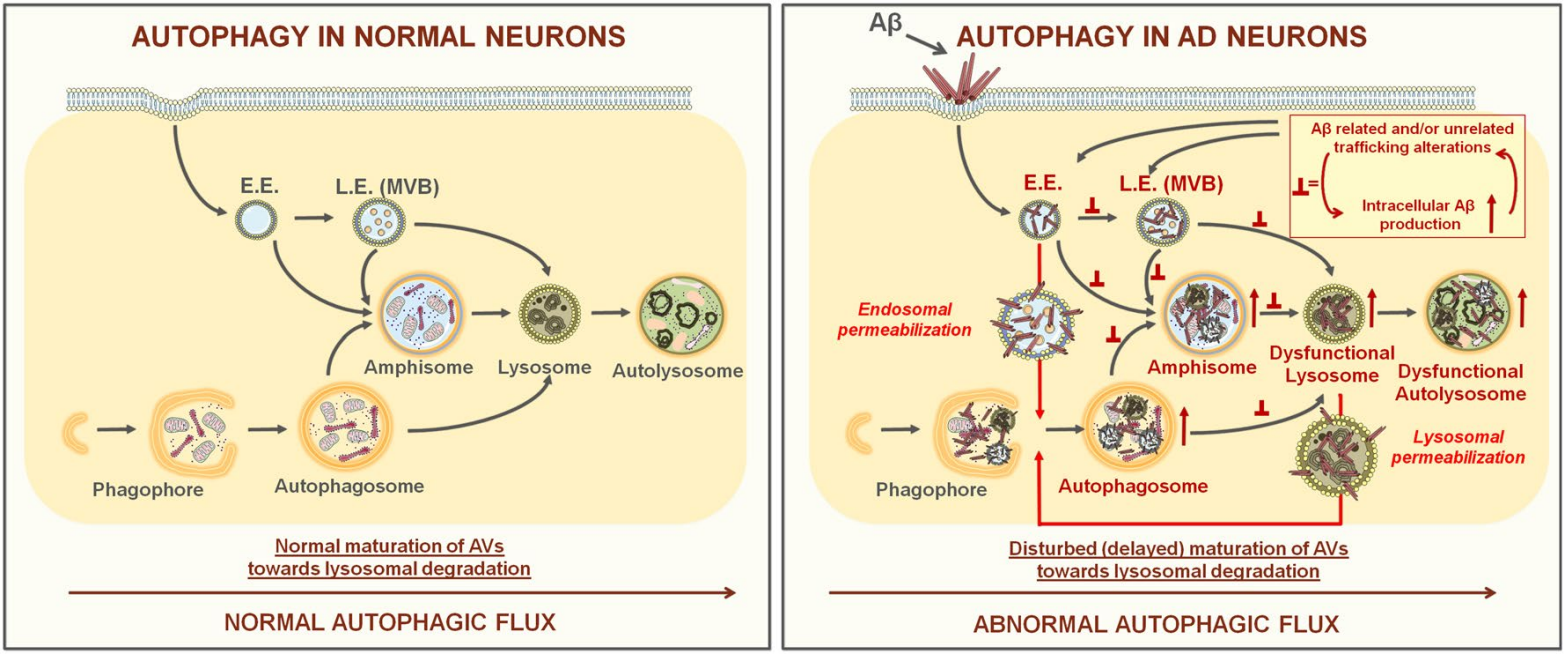
Stepwise autophagy progression in normal and AD-affected neurons. *Left* different steps in autophagosome formation and maturation under normal (physiological) conditions, starting from phagophore expansion. Before fusing with lysosomes, double-membraned autophagosomes can also merge with early (E.E) and late endosomes (L.E./MVBs) giving rise to amphisomes. *Right* in AD-related processes, normal autophagic flux is compromised. This results in a pile up of autophagic vacuoles (autophagosomes, amphisomes, autolysosomes) due to disturbed trafficking, fusion with and/or degradation processes within dysfunctional lysosomes. These changes are particularly pronounced in dystrophic neurites, where they likely contribute to synapto-/neurotoxicity (see also Fig. 1c). Here, intracellular toxic Aβ species may work together with dysfunctional endosomal sorting/trafficking mechanisms in aggravating these degradative abnormalities. *Inset* in the *right panel* depicts this hypothesized self-propelling endosomal dysfunction, while the *arrows* from it point toward the likely sites where consequent disturbances in membrane flow “roadblocks” may occur. These pathogenic processes may eventually compromise the impermeability of endolysosomal compartments. Because autophagy can directly target toxic Aβ species [62] as well as injured lysosomes [80], we hypothesize that in the AD context both physically injured endosomes/lysosomes and toxic Aβ species released into cytosol may become its targets. Here, initially autophagy may be protective, but as the disease develops and the toxic burden exceeds cellular reparative capacity, neuronal death may follow

Autophagy-mediated degradation is particularly important in neurons because these postmitotic cells are otherwise unable to dilute accumulating toxic cytoplasmic debris and dysfunctional organelles (e.g., mitochondria) through subsequent cellular divisions. It is therefore not surprising that defects in the highly efficient baseline autophagic flux, likely at later AV maturation stages, are relevant to AD pathogenesis [14] (Fig. 4).

Importantly, studies from the last two decades established that undisturbed autophagic flux requires a tight cooperation between the endosomal compartments and AVs. For instance, before fusing with lysosomes and forming autolysosomes, autophagosomes can also directly fuse with early and/or late endosomes, to form hybrid structures named amphisomes [9, 75] (Fig. 4). Moreover, successful autophagic degradation not only requires undisturbed early and late endosomal sorting/maturation [40, 114, 121], but autophagosomal biogenesis mechanisms also share some regulators with endosomal compartments [64, 72] and may even rely on recycling endosomes as a source of membranes [110].

Indeed, key endosomal recycling regulators appear to be functionally involved at crossing points of these two cellular pathways. For instance Rab11, in addition to facilitating the physical merger of MVBs with autophagosomes [39, 143], also functions in early steps of autophagosome formation [77]. On the other hand, a recent study revealed a novel role for ARF6 in late endosomal maturation (sorting) [44], which, similarly to Rab11, in addition to cellular recycling also promotes biogenesis of autophagosomes [59, 92]. Interestingly, these roles of ARF6 are mediated via its downstream effector phospholipase D (PLD) [59, 92], an enzyme which as well takes part in the later maturation steps of autophagy [25]. Here, PLD seems to operate downstream of the class III phosphatidylinositol-3 kinase (hereafter referred to as PI3K) [25], a multimeric protein complex and a key regulator of both autophagy and endosomal trafficking [42, 112]. PI3K drives the phosphorylation of phosphatidylinositol (PI) to produce PI3P, a membrane-localized lipid species that recruits proteins with specific binding modules and distinct regulatory functions in endosomal trafficking/signaling and/or autophagy [70, 103, 112]. The catalytic component of the PI3K was originally identified in yeast as a regulator of vesicular protein targeting to the vacuole (analogous process to endolysosomal protein trafficking in mammals), and named therefore vacuolar protein sorting 34 (Vps34) [129]. Later studies revealed the human orthologs of both Vps34 (PIK3C3) and one of its regulatory subunits Vps15 (p150/PIK3R4) [106, 158]. Interestingly, Vps34 may affect autophagy at its early, initiating stages, via direct involvement in autophagosomal biogenesis, as well as at its later maturation steps [42]. Thereby, the cellular activities of Vps34 resemble the above-mentioned small GTPases (Rab11/ARF6); nevertheless, how exactly these proteins are functionally related remains to be established. We do however know that in these dual and converging PI3K activities in autophagy, particularly important is the role of several proteins associated with beclin 1, which either alone or as part of a PI3K/beclin 1 core complex affect these specific processes [49]. Beclin 1 is a mammalian ortholog of the yeast autophagy-related 6 (Atg6)/Vps30 protein and an essential component of the PI3 K complex, important in various (patho)physiological processes, including neurodegeneration [49]. Its functional/physical interactions within the PI3K protein complex are of pivotal relevance for cellular homeostasis, as they ensure dynamic coordination of specific endosomal trafficking and autophagy steps at various levels [72, 86, 120].

In both yeast and mammals, two distinct mutually exclusive beclin 1 containing PI3K complexes exist. The first one contains the yeast Atg14 [mammalian Atg14L (yeast Atg14-like)/Beclin 1-associated autophagy-related key regulator (Barcor)] protein [53, 63, 140]. Conversely, the other complex contains only the Vps38 protein [53, 63], a likely functional analog of the mammalian UV irradiation resistance-associated gene (UVRAG) [54]. In yeast, Atg14 and Vps38 complexes have specific subcellular localization and distinct functions in early autophagosomal biogenesis and protein transport to the vacuole, respectively [63, 104]. While a similar functional specification has been implied also for their mammalian counterparts [53, 54, 148], a recent study demonstrated that here Atg14L as well may affect the endocytic trafficking (independently of its association with beclin 1), underscoring its more complex regulatory functions in higher organisms [64]. This higher complexity is further highlighted by the function of another PI3K/beclin 1 complex component not present in yeast, namely RUN domain and cysteine-rich domain containing beclin 1-interacting protein (Rubicon). Rubicon interacts with a subpopulation of UVRAG containing PI3K/beclin 1 complexes [86] and blocks the autophagosomal maturation steps and autophagosomal clearance in lysosomes, by directly inhibiting PI3K activity and other mechanisms involved in endosomal/autophagosomal maturation [86, 141, 142]. Taken together, these findings highlight the fact that different roles of PI3K/beclin 1 complex may relate to specific accessory proteins which at particular subcellular sites synchronize the functioning of autophagic and the endolysosomal system to ensure undisturbed degradation of incoming cargo from both pathways.

### PI3K/beclin 1 complex and its relevance in AD

These mechanisms may be of great importance in AD, as decreased PI3P, Vps34 and beclin 1 levels have all been reported [57, 93, 107]. Importantly, as genetic ablation of beclin 1 or Vps34 results in reduced levels of both Atg14L and UVRAG [53, 148], these changes in addition to compromising autophagy as well may affect endolysosomal trafficking [148]. Indeed, the phenotypes of beclin 1- and Vps34-deficient mice are more severe (E7.5–8.5; lethal) [173, 176] than that of other autophagy-related genes, such as Atg3 [136], Atg5 [67] and Atg16L [124] (P1; neonatal lethal). This suggests additional, autophagy-independent cellular roles of beclin 1 and Vps34, some of which likely relate to their endolysosomal trafficking function.

In line, Vps34 deficiency in sensory neurons leads to rapid neurodegeneration, primarily resulting from disruption of the endosomal and not the autophagic pathway [177]. Similarly, Vps34 downregulation in primary cortical neurons results in impaired endosomal sorting and consequent endosomal swelling [93].

Also in relation to beclin 1, recent studies demonstrate that this protein may not be exclusively involved in autophagy initiation, as originally proposed [174], but as well could play a role in endocytic trafficking regulation. For instance, beclin 1, Vps34, Vps15 and UVRAG were all shown to take part in trafficking of membrane receptors toward lysosomes [148]. Moreover, in *C. elegans* both beclin 1 ortholog (BEC-1) and Vps34 are pivotal in retrograde retromer-mediated endocytic sorting toward the TGN [118]. Finally, a similar role in endocytic trafficking for beclin1 has also been demonstrated in *Drosophila* [134].

In beclin 1^+/−^ mice also lysosomal abnormalities are noted, while their crossing with an APP-based AD mouse model results in higher intraneuronal and extraneuronal Aβ levels, more profound ultrastructural defects, including more severe lysosomal/autophagic abnormalities as well as increased neurodegeneration [107]. Beclin 1 overexpression in the same AD model reduces intraneuronal Aβ and extracellular plaque pathology [107], thus underscoring that turnover and/or excessive production of toxic Aβ peptides may rely on beclin 1-mediated functions. To this end, in the follow-up study, the authors show that beclin 1 transient downregulation in parallel to increasing the Aβ secretion also causes intracellular accumulation of APP and APP-CTFs [57]. Interestingly however, in beclin 1 silenced cells and in AD brains where beclin 1 and Vps34 are reduced, higher levels of autophagosomal marker LC3-II (the lipidated form of the microtubule-associated protein 1 light chain 3) are also observed [57]. As this indicates boosted (not hampered) autophagy, which would be expected should beclin 1 exclusively regulate autophagy initiation, to explain their findings Jaeger and colleagues proposed that beclin 1 may also regulate later maturation steps of autophagy. Here, defective beclin 1-dependent clearance of autophagic compartments would explain the increased levels of APP metabolites and Aβ. These findings are also consistent with a primary defect in the endolysosomal trafficking pathway: in the study of Jaeger et al., lowered beclin 1 levels parallel those of Vps34 not only in AD, but also in cells where either of these two proteins is silenced [57]. This is important, as in AD lowered levels of Vps34 and its product PI3P may disturb normal endosomal sorting of APP, thus causing its enhanced amyloidogenic processing [93]. As decreased PI3P levels also directly impact functioning of the endosomal sorting complex required for transport (ESCRT) [93], which is pivotal in MVB maturation as well as fusion of AVs with the endolysosomal system and consequent cargo degradation [121], a primary defect in the endolysosomal system provides an alternative explanation for the observed effects by Jaeger and collaegues [57].

In line, although AVs were implied as an important source of amyloidogenic activity in neurons [172], more recently Boland et al. contested this view by showing a more likely primary role of the endolysosomal system in APP processing [15]. Despite these conceptual disparities, both studies, however, largely agree with respect to the contribution of disturbed lysosomal degradation in these phenomena [15, 172]. Accordingly, in TgCRND8 AD mice, improved lysosomal degradative function, achieved through genetic ablation of endogenous lysosomal cysteine protease inhibitor cystatin B, rescues autophagic/lysosomal dysfunction and amyloid pathology, as well as related memory and cognitive deficits [170].

Taken together, all these findings further strengthen the important role of the endolysosomal system in amyloidogenesis and balanced autophagic degradation (Fig. 4).

### Granulovacuolar degeneration (GVD) bodies: additional pathomorphological link between the endolysosomal and autophagy dysfunction in AD

Endolysosomal and autophagy dysfunctions in AD may also be linked via an underappreciated pathomorphological feature of this disease, namely the granulovacuolar degeneration (GVD) bodies. These intracellular, double membrane-bound organelles, with electron-dense core granules [105], occur in relation to aging and different neurodegenerative disorders, but only in AD do these autophagic-like structures [43] disseminate in an orderly hierarchical pattern, which correlates with distribution of several disease progression markers and the degree of dementia [145].

Interestingly, GVD bodies stain positively for the charged multi-vesicular body protein 2B (CHMP2B) [43]. This subunit of the ESCRT-III protein complex, involved in intraluminal vesicle (ILV) sorting in MVBs, is important in successful autophagic degradation, as *CHMP2B* mutations, found in a subset of FTD and amyotrophic later sclerosis (ALS) patients, compromise lysosomal degradation of AV cargo [40]. In line, depletion of other ESCRT complex components produces a similar phenotype. Here, autophagosomes and amphisomes seem to be normally formed; however, ESCRT depletion-related sorting defects impair fusion of these AVs with lysosomes and thus degradation in nascent autolysosomes [40]. Based on their resemblance to late-stage AVs, CHMP2B-positive GVD bodies were also proposed to accumulate due to a failure in autolysosome formation [43].

Interestingly, GVD bodies may also link to tau pathology. Accordingly, several prominent tau kinases, such as casein kinase 1 delta (CK1δ) [61], glycogen synthase kinase-3 beta (GSK3β) [71] and cyclin-dependent protein kinase-5 (CDK5) [100], physically associate with GVD bodies, which appear in correlation with the early accumulation of phospho-tau pathology [168]. This provides additional proof for the concept proposed by us that aberrant tau phosphorylation may in fact stem from endolysosomal–autophagic deficits and related cellular signaling and/or degradative abnormalities (see previously and Fig. 1c).

GVD bodies also contain lipid raft marker proteins, such as flotillin-1 [100]. In this respect, we reported that the PSEN1-interactor ICAM-5 associates with flotillin-1 both at the cell surface and within endosomal compartments [111]. Moreover, in “aged” (in vitro) WT primary neurons, ICAM-5 accumulations (similar to those observed in younger PSEN1^−/−^ neurons [37]), much like GVD bodies, stain positively for flotillin-1 as well as late endosomal MVBs [111]. These convergent pathomorphological and cell biological findings may reflect commonalities in endolysosomal dysfunction that occur in relation to aging and of relevance to AD. Here, underlying transport jamming and/or delayed cargo degradation potentially relates to either declining function (levels) of PSEN1 or another relevant trafficking deficit. Taken together, all this further strengthens the notion that autophagic and endolysosomal trafficking pathways cannot be perceived as autonomous, physically separate entities, but rather as two functional components of an integrated cellular system, the balance of which in AD may become compromised at various levels and in relation to many different factors.

## Conclusions

We here provide a comprehensive literature overview to highlight the important role of endolysosomal trafficking as well as autophagy in pathogenic processes underlying AD. Overall, the available data strongly argue that in AD, defective endosomal sorting/trafficking and lysosomal dysfunction may work together with intracellular (endosomal) Aβ accumulation to subsequently affect the late autophagy stages, leading to inefficient clearance of AVs and thus resulting in their progressive buildup (Fig. 4). This is supported by the fact that autophagic degradation requires undisturbed endolysosomal sorting and that in unrelated neurodegenerative diseases, like NPC, similar autophagic phenotypes result from primary endolysosomal deficits. Following the same analogy to NPC, we also hypothesize that in AD these pathogenic mechanisms may as well contribute to abnormal tau phosphorylation and accumulation of toxic tau species.

Considering the multistep character of the autophagic process and its major reliance on endolysosomal trafficking regulation, therapeutic strategies aiming at promoting autophagic activity in AD will most likely have to be combined with treatments which would concomitantly enhance the performance of lysosomal degradation to allow efficient turnover of the incoming AVs. Transcription factor EB (TFEB) fulfills both of these criteria as it coordinately activates lysosomal biogenesis as well as genes required for autophagosomal formation [132]. As its efficacy has already been demonstrated in several diseases, including lysosomal storage disorders [137], Huntington’s disease (HD) [152] and Parkinson’s disease (PD) [29], it is expected that similar benefits may also be achieved in the AD context. To this end, a recently published study provides a first support that TFEB may indeed be beneficial in AD and other tauopathies [109]. Another way to tackle the disturbed lysosomal function and AV clearance may involve pharmacological treatments which would improve the catalytic performance of lysosomal enzymes, as implied by the study of Yang et al. [170]. Alternatively, interventions aiming at alleviating the burden to the endolysosomal compartments causing their inappropriate functioning as well hold some potential. Here, for instance, lowering cholesterol and/or preventing Aβ production/oligomerization may all prove beneficial. Indeed, in both NPC and AD, the cholesterol-lowering drug 2-hydroxypropyl-beta-cyclodextrin (HP-β-cyclodextrin) is emerging as a potentially useful pharmacological tool [4, 171]. In light of Aβ in turn, our recent work implies that peptides that disrupt the physical interaction between the APP and PSEN1 may be useful selective inhibitors of Aβ production [38]. Finally, as growing evidence suggests that restoring proper endosomal trafficking (recycling) may be similarly efficient, development of specific pharmacological modulators of these processes may constitute another potential strategy. Here, a recently developed pharmacological stabilizer of the retromer sorting complex provides a first proof of concept [89]. Indeed, given the relatively early character of endolysosomal/dysfunction in AD, and a major reliance of amyloidogenic processing on sorting regulators, future therapeutic efforts should maybe aim to lengthen the fidelity of endosomal transport and degradation, and not only focused on majorly targeting the amyloidogenic enzymes, BACE1 and γ-secretase complexes.

## References

[1] Almeida CG, Takahashi RH, Gouras GK (2006). Beta-amyloid accumulation impairs multivesicular body sorting by inhibiting the ubiquitin-proteasome system. J Neurosci.

[2] Alonso AC, Grundke-Iqbal I, Iqbal K (1996). Alzheimer’s disease hyperphosphorylated tau sequesters normal tau into tangles of filaments and disassembles microtubules. Nat Med.

[3] Annaert WG, Esselens C, Baert V, Boeve C, Snellings G, Cupers P, Craessaerts K, De Strooper B (2001). Interaction with telencephalin and the amyloid precursor protein predicts a ring structure for presenilins. Neuron.

[4] Aqul A, Liu B, Ramirez CM, Pieper AA, Estill SJ, Burns DK, Repa JJ, Turley SD, Dietschy JM (2011). Unesterified cholesterol accumulation in late endosomes/lysosomes causes neurodegeneration and is prevented by driving cholesterol export from this compartment. J Neurosci.

[5] Arriagada PV, Growdon JH, Hedley-Whyte ET, Hyman BT (1992). Neurofibrillary tangles but not senile plaques parallel duration and severity of Alzheimer’s disease. Neurology.

[6] Baki L, Neve RL, Shao Z, Shioi J, Georgakopoulos A, Robakis NK (2008). Wild-type but not FAD mutant presenilin-1 prevents neuronal degeneration by promoting phosphatidylinositol 3-kinase neuroprotective signaling. J Neurosci.

[7] Baki L, Shioi J, Wen P, Shao Z, Schwarzman A, Gama-Sosa M, Neve R, Robakis NK (2004). PS1 activates PI3K thus inhibiting GSK-3 activity and tau overphosphorylation: effects of FAD mutations. EMBO J.

[8] Ballatore C, Lee VM, Trojanowski JQ (2007). Tau-mediated neurodegeneration in Alzheimer’s disease and related disorders. Nat Rev Neurosci.

[9] Berg TO, Fengsrud M, Stromhaug PE, Berg T, Seglen PO (1998). Isolation and characterization of rat liver amphisomes. Evidence for fusion of autophagosomes with both early and late endosomes. J Biol Chem.

[10] Bhaskar K, Yen SH, Lee G (2005). Disease-related modifications in tau affect the interaction between Fyn and Tau. J Biol Chem.

[11] Billings LM, Oddo S, Green KN, McGaugh JL, LaFerla FM (2005). Intraneuronal Abeta causes the onset of early Alzheimer’s disease-related cognitive deficits in transgenic mice. Neuron.

[12] Binder LI, Frankfurter A, Rebhun LI (1985). The distribution of tau in the mammalian central nervous system. J Cell Biol.

[13] Blennow K, de Leon MJ, Zetterberg H (2006). Alzheimer’s disease. Lancet.

[14] Boland B, Kumar A, Lee S, Platt FM, Wegiel J, Yu WH, Nixon RA (2008). Autophagy induction and autophagosome clearance in neurons: relationship to autophagic pathology in Alzheimer’s disease. J Neurosci.

[15] Boland B, Smith DA, Mooney D, Jung SS, Walsh DM, Platt FM (2010). Macroautophagy is not directly involved in the metabolism of amyloid precursor protein. J Biol Chem.

[16] Braak H, Alafuzoff I, Arzberger T, Kretzschmar H, Del Tredici K (2006). Staging of Alzheimer disease-associated neurofibrillary pathology using paraffin sections and immunocytochemistry. Acta Neuropathol.

[17] Caglayan S, Takagi-Niidome S, Liao F, Carlo AS, Schmidt V, Burgert T, Kitago Y, Fuchtbauer EM, Fuchtbauer A, Holtzman DM, Takagi J, Willnow TE (2014). Lysosomal sorting of amyloid-beta by the SORLA receptor is impaired by a familial Alzheimer’s disease mutation. Sci Transl Med.

[18] Cairns NJ, Bigio EH, Mackenzie IR, Neumann M, Lee VM, Hatanpaa KJ, White CL, Schneider JA, Grinberg LT, Halliday G, Duyckaerts C, Lowe JS, Holm IE, Tolnay M, Okamoto K, Yokoo H, Murayama S, Woulfe J, Munoz DG, Dickson DW, Ince PG, Trojanowski JQ, Mann DM (2007). Neuropathologic diagnostic and nosologic criteria for frontotemporal lobar degeneration: consensus of the Consortium for Frontotemporal Lobar Degeneration. Acta Neuropathol.

[19] Cataldo AM, Petanceska S, Terio NB, Peterhoff CM, Durham R, Mercken M, Mehta PD, Buxbaum J, Haroutunian V, Nixon RA (2004). Abeta localization in abnormal endosomes: association with earliest Abeta elevations in AD and Down syndrome. Neurobiol Aging.

[20] Cataldo AM, Peterhoff CM, Schmidt SD, Terio NB, Duff K, Beard M, Mathews PM, Nixon RA (2004). Presenilin mutations in familial Alzheimer disease and transgenic mouse models accelerate neuronal lysosomal pathology. J Neuropathol Exp Neurol.

[21] Chaineau M, Ioannou MS, McPherson PS (2013). Rab35: GEFs. GAPs and effectors. Traffic.

[22] Chavez-Gutierrez L, Bammens L, Benilova I, Vandersteen A, Benurwar M, Borgers M, Lismont S, Zhou L, Van Cleynenbreugel S, Esselmann H, Wiltfang J, Serneels L, Karran E, Gijsen H, Schymkowitz J, Rousseau F, Broersen K, De Strooper B (2012). The mechanism of gamma-Secretase dysfunction in familial Alzheimer disease. EMBO J.

[23] Christensen DZ, Kraus SL, Flohr A, Cotel MC, Wirths O, Bayer TA (2008). Transient intraneuronal A beta rather than extracellular plaque pathology correlates with neuron loss in the frontal cortex of APP/PS1KI mice. Acta Neuropathol.

[24] Coen K, Flannagan RS, Baron S, Carraro-Lacroix LR, Wang D, Vermeire W, Michiels C, Munck S, Baert V, Sugita S, Wuytack F, Hiesinger PR, Grinstein S, Annaert W (2012). Lysosomal calcium homeostasis defects, not proton pump defects, cause endo-lysosomal dysfunction in PSEN-deficient cells. J Cell Biol.

[25] Dall’Armi C, Hurtado-Lorenzo A, Tian H, Morel E, Nezu A, Chan RB, Yu WH, Robinson KS, Yeku O, Small SA, Duff K, Frohman MA, Wenk MR, Yamamoto A, Di Paolo G (2010). The phospholipase D1 pathway modulates macroautophagy. Nat Commun.

[26] Damme M, Suntio T, Saftig P, Eskelinen EL (2014) Autophagy in neuronal cells: general principles and physiological and pathological functions. Acta Neuropathol. doi:10.1007/s00401-014-1361-4 10.1007/s00401-014-1361-4 25367385

[27] Dawson HN, Cantillana V, Jansen M, Wang H, Vitek MP, Wilcock DM, Lynch JR, Laskowitz DT (2010). Loss of tau elicits axonal degeneration in a mouse model of Alzheimer’s disease. Neuroscience.

[28] De Strooper B, Annaert W (2010). Novel research horizons for presenilins and gamma-secretases in cell biology and disease. Annu Rev Cell Dev Biol.

[29] Decressac M, Mattsson B, Weikop P, Lundblad M, Jakobsson J, Bjorklund A (2013). TFEB-mediated autophagy rescues midbrain dopamine neurons from alpha-synuclein toxicity. Proc Natl Acad Sci USA.

[30] Desikan RS, McEvoy LK, Thompson WK, Holland D, Brewer JB, Aisen PS, Sperling RA, Dale AM (2012). Amyloid-beta–associated clinical decline occurs only in the presence of elevated P-tau. Arch Neurol.

[31] Ditaranto K, Tekirian TL, Yang AJ (2001). Lysosomal membrane damage in soluble Abeta-mediated cell death in Alzheimer’s disease. Neurobiol Dis.

[32] Dixit R, Ross JL, Goldman YE, Holzbaur EL (2008). Differential regulation of dynein and kinesin motor proteins by tau. Science.

[33] Dobrowolski R, Vick P, Ploper D, Gumper I, Snitkin H, Sabatini DD, De Robertis EM (2012). Presenilin deficiency or lysosomal inhibition enhances Wnt signaling through relocalization of GSK3 to the late-endosomal compartment. Cell Rep.

[34] Dodson SE, Andersen OM, Karmali V, Fritz JJ, Cheng D, Peng J, Levey AI, Willnow TE, Lah JJ (2008). Loss of LR11/SORLA enhances early pathology in a mouse model of amyloidosis: evidence for a proximal role in Alzheimer’s disease. J Neurosci.

[35] Dumanchin C, Czech C, Campion D, Cuif MH, Poyot T, Martin C, Charbonnier F, Goud B, Pradier L, Frebourg T (1999). Presenilins interact with Rab11, a small GTPase involved in the regulation of vesicular transport. Hum Mol Genet.

[36] Elrick MJ, Yu T, Chung C, Lieberman AP (2012). Impaired proteolysis underlies autophagic dysfunction in Niemann-Pick type C disease. Hum Mol Genet.

[37] Esselens C, Oorschot V, Baert V, Raemaekers T, Spittaels K, Serneels L, Zheng H, Saftig P, De Strooper B, Klumperman J, Annaert W (2004). Presenilin 1 mediates the turnover of telencephalin in hippocampal neurons via an autophagic degradative pathway. J Cell Biol.

[38] Esselens C, Sannerud R, Gallardo R, Baert V, Kaden D, Serneels L, De Strooper B, Rousseau F, Multhaup G, Schymkowitz J, Langedijk JP, Annaert W (2012). Peptides based on the presenilin-APP binding domain inhibit APP processing and Abeta production through interfering with the APP transmembrane domain. FASEB J.

[39] Fader CM, Sanchez D, Furlan M, Colombo MI (2008). Induction of autophagy promotes fusion of multivesicular bodies with autophagic vacuoles in k562 cells. Traffic.

[40] Filimonenko M, Stuffers S, Raiborg C, Yamamoto A, Malerod L, Fisher EM, Isaacs A, Brech A, Stenmark H, Simonsen A (2007). Functional multivesicular bodies are required for autophagic clearance of protein aggregates associated with neurodegenerative disease. J Cell Biol.

[41] Friedrich RP, Tepper K, Ronicke R, Soom M, Westermann M, Reymann K, Kaether C, Fandrich M (2010). Mechanism of amyloid plaque formation suggests an intracellular basis of Abeta pathogenicity. Proc Natl Acad Sci USA.

[42] Funderburk SF, Wang QJ, Yue Z (2010). The Beclin 1-VPS34 complex–at the crossroads of autophagy and beyond. Trends Cell Biol.

[43] Funk KE, Mrak RE, Kuret J (2011). Granulovacuolar degeneration (GVD) bodies of Alzheimer’s disease (AD) resemble late-stage autophagic organelles. Neuropathol Appl Neurobiol.

[44] Ghossoub R, Lembo F, Rubio A, Gaillard CB, Bouchet J, Vitale N, Slavik J, Machala M, Zimmermann P (2014). Syntenin-ALIX exosome biogenesis and budding into multivesicular bodies are controlled by ARF6 and PLD2. Nat Commun.

[45] Gomez-Isla T, Hollister R, West H, Mui S, Growdon JH, Petersen RC, Parisi JE, Hyman BT (1997). Neuronal loss correlates with but exceeds neurofibrillary tangles in Alzheimer’s disease. Ann Neurol.

[46] Gotz J, Chen F, van Dorpe J, Nitsch RM (2001). Formation of neurofibrillary tangles in P301 l tau transgenic mice induced by Abeta 42 fibrils. Science.

[47] Gotz J, Xia D, Leinenga G, Chew YL, Nicholas H (2013). What renders TAU toxic. Front Neurol.

[48] He C, Klionsky DJ (2009). Regulation mechanisms and signaling pathways of autophagy. Annu Rev Genet.

[49] He C, Levine B (2010). The Beclin 1 interactome. Curr Opin Cell Biol.

[50] Hoover BR, Reed MN, Su J, Penrod RD, Kotilinek LA, Grant MK, Pitstick R, Carlson GA, Lanier LM, Yuan LL, Ashe KH, Liao D (2010). Tau mislocalization to dendritic spines mediates synaptic dysfunction independently of neurodegeneration. Neuron.

[51] Hu X, Crick SL, Bu G, Frieden C, Pappu RV, Lee JM (2009). Amyloid seeds formed by cellular uptake, concentration, and aggregation of the amyloid-beta peptide. Proc Natl Acad Sci USA.

[52] Inoue K, Rispoli J, Kaphzan H, Klann E, Chen EI, Kim J, Komatsu M, Abeliovich A (2012). Macroautophagy deficiency mediates age-dependent neurodegeneration through a phospho-tau pathway. Mol Neurodegener.

[53] Itakura E, Kishi C, Inoue K, Mizushima N (2008). Beclin 1 forms two distinct phosphatidylinositol 3-kinase complexes with mammalian Atg14 and UVRAG. Mol Biol Cell.

[54] Itakura E, Mizushima N (2009). Atg14 and UVRAG: mutually exclusive subunits of mammalian Beclin 1-PI3K complexes. Autophagy.

[55] Ittner LM, Ke YD, Delerue F, Bi M, Gladbach A, van Eersel J, Wolfing H, Chieng BC, Christie MJ, Napier IA, Eckert A, Staufenbiel M, Hardeman E, Gotz J (2010). Dendritic function of tau mediates amyloid-beta toxicity in Alzheimer’s disease mouse models. Cell.

[56] Ittner LM, Ke YD, Gotz J (2009). Phosphorylated Tau interacts with c-Jun N-terminal kinase-interacting protein 1 (JIP1) in Alzheimer disease. J Biol Chem.

[57] Jaeger PA, Pickford F, Sun CH, Lucin KM, Masliah E, Wyss-Coray T (2010). Regulation of amyloid precursor protein processing by the Beclin 1 complex. PLoS One.

[58] Jin LW, Shie FS, Maezawa I, Vincent I, Bird T (2004). Intracellular accumulation of amyloidogenic fragments of amyloid-beta precursor protein in neurons with Niemann-Pick type C defects is associated with endosomal abnormalities. Am J Pathol.

[59] Jovanovic OA, Brown FD, Donaldson JG (2006). An effector domain mutant of Arf6 implicates phospholipase D in endosomal membrane recycling. Mol Biol Cell.

[60] Jurisch-Yaksi N, Sannerud R, Annaert W (2013). A fast growing spectrum of biological functions of gamma-secretase in development and disease. Biochim Biophys Acta.

[61] Kannanayakal TJ, Tao H, Vandre DD, Kuret J (2006). Casein kinase-1 isoforms differentially associate with neurofibrillary and granulovacuolar degeneration lesions. Acta Neuropathol.

[62] Khandelwal PJ, Herman AM, Hoe HS, Rebeck GW, Moussa CE (2011). Parkin mediates beclin-dependent autophagic clearance of defective mitochondria and ubiquitinated Abeta in AD models. Hum Mol Genet.

[63] Kihara A, Noda T, Ishihara N, Ohsumi Y (2001). Two distinct Vps34 phosphatidylinositol 3-kinase complexes function in autophagy and carboxypeptidase Y sorting in *Saccharomyces cerevisiae*. J Cell Biol.

[64] Kim HJ, Zhong Q, Sheng ZH, Yoshimori T, Liang C, Jung JU (2012). Beclin-1-interacting autophagy protein Atg14L targets the SNARE-associated protein Snapin to coordinate endocytic trafficking. J Cell Sci.

[65] Knobloch M, Konietzko U, Krebs DC, Nitsch RM (2007). Intracellular Abeta and cognitive deficits precede beta-amyloid deposition in transgenic arcAbeta mice. Neurobiol Aging.

[66] Kroemer G, Marino G, Levine B (2010). Autophagy and the integrated stress response. Mol Cell.

[67] Kuma A, Hatano M, Matsui M, Yamamoto A, Nakaya H, Yoshimori T, Ohsumi Y, Tokuhisa T, Mizushima N (2004). The role of autophagy during the early neonatal starvation period. Nature.

[68] Lane RF, Steele JW, Cai D, Ehrlich ME, Attie AD, Gandy S (2013). Protein sorting motifs in the cytoplasmic tail of SorCS1 control generation of Alzheimer’s amyloid-beta peptide. J Neurosci.

[69] Lee JH, Yu WH, Kumar A, Lee S, Mohan PS, Peterhoff CM, Wolfe DM, Martinez-Vicente M, Massey AC, Sovak G, Uchiyama Y, Westaway D, Cuervo AM, Nixon RA (2010). Lysosomal proteolysis and autophagy require presenilin 1 and are disrupted by Alzheimer-related PS1 mutations. Cell.

[70] Leevers SJ, Vanhaesebroeck B, Waterfield MD (1999). Signalling through phosphoinositide 3-kinases: the lipids take centre stage. Curr Opin Cell Biol.

[71] Leroy K, Boutajangout A, Authelet M, Woodgett JR, Anderton BH, Brion JP (2002). The active form of glycogen synthase kinase-3beta is associated with granulovacuolar degeneration in neurons in Alzheimer’s disease. Acta Neuropathol.

[72] Liang C, Lee JS, Inn KS, Gack MU, Li Q, Roberts EA, Vergne I, Deretic V, Feng P, Akazawa C, Jung JU (2008). Beclin1-binding UVRAG targets the class C Vps complex to coordinate autophagosome maturation and endocytic trafficking. Nat Cell Biol.

[73] Liao G, Yao Y, Liu J, Yu Z, Cheung S, Xie A, Liang X, Bi X (2007). Cholesterol accumulation is associated with lysosomal dysfunction and autophagic stress in Npc1^−/−^ mouse brain. Am J Pathol.

[74] Ling D, Song HJ, Garza D, Neufeld TP, Salvaterra PM (2009). Abeta42-induced neurodegeneration via an age-dependent autophagic-lysosomal injury in Drosophila. PLoS One.

[75] Liou W, Geuze HJ, Geelen MJ, Slot JW (1997). The autophagic and endocytic pathways converge at the nascent autophagic vacuoles. J Cell Biol.

[76] Lloyd-Evans E, Morgan AJ, He X, Smith DA, Elliot-Smith E, Sillence DJ, Churchill GC, Schuchman EH, Galione A, Platt FM (2008). Niemann-Pick disease type C1 is a sphingosine storage disease that causes deregulation of lysosomal calcium. Nat Med.

[77] Longatti A, Lamb CA, Razi M, Yoshimura S, Barr FA, Tooze SA (2012). TBC1D14 regulates autophagosome formation via Rab11- and ULK1-positive recycling endosomes. J Cell Biol.

[78] Love S, Bridges LR, Case CP (1995). Neurofibrillary tangles in Niemann-Pick disease type C. Brain.

[79] Lue LF, Kuo YM, Roher AE, Brachova L, Shen Y, Sue L, Beach T, Kurth JH, Rydel RE, Rogers J (1999). Soluble amyloid beta peptide concentration as a predictor of synaptic change in Alzheimer’s disease. Am J Pathol.

[80] Maejima I, Takahashi A, Omori H, Kimura T, Takabatake Y, Saitoh T, Yamamoto A, Hamasaki M, Noda T, Isaka Y, Yoshimori T (2013). Autophagy sequesters damaged lysosomes to control lysosomal biogenesis and kidney injury. EMBO J.

[81] Malnar M, Kosicek M, Lisica A, Posavec M, Krolo A, Njavro J, Omerbasic D, Tahirovic S, Hecimovic S (2012). Cholesterol-depletion corrects APP and BACE1 misstrafficking in NPC1-deficient cells. Biochim Biophys Acta.

[82] Martin L, Latypova X, Wilson CM, Magnaudeix A, Perrin ML, Terro F (2013). Tau protein phosphatases in Alzheimer’s disease: the leading role of PP2A. Ageing Res Rev.

[83] Martin L, Latypova X, Wilson CM, Magnaudeix A, Perrin ML, Yardin C, Terro F (2013). Tau protein kinases: involvement in Alzheimer’s disease. Ageing Res Rev.

[84] Martinez-Lopez N, Athonvarangkul D, Mishall P, Sahu S, Singh R (2013). Autophagy proteins regulate ERK phosphorylation. Nat Commun.

[85] Martinez-Lopez N, Singh R (2014). ATGs: Scaffolds for MAPK/ERK signaling. Autophagy.

[86] Matsunaga K, Saitoh T, Tabata K, Omori H, Satoh T, Kurotori N, Maejima I, Shirahama-Noda K, Ichimura T, Isobe T, Akira S, Noda T, Yoshimori T (2009). Two Beclin 1-binding proteins, Atg14L and Rubicon, reciprocally regulate autophagy at different stages. Nat Cell Biol.

[87] Mc Donald JM, Savva GM, Brayne C, Welzel AT, Forster G, Shankar GM, Selkoe DJ, Ince PG, Walsh DM (2010). The presence of sodium dodecyl sulphate-stable Abeta dimers is strongly associated with Alzheimer-type dementia. Brain.

[88] McLean CA, Cherny RA, Fraser FW, Fuller SJ, Smith MJ, Beyreuther K, Bush AI, Masters CL (1999). Soluble pool of Abeta amyloid as a determinant of severity of neurodegeneration in Alzheimer’s disease. Ann Neurol.

[89] Mecozzi VJ, Berman DE, Simoes S, Vetanovetz C, Awal MR, Patel VM, Schneider RT, Petsko GA, Ringe D, Small SA (2014). Pharmacological chaperones stabilize retromer to limit APP processing. Nat Chem Biol.

[90] Miaczynska M, Pelkmans L, Zerial M (2004). Not just a sink: endosomes in control of signal transduction. Curr Opin Cell Biol.

[91] Mohamed A, Saavedra L, Di Pardo A, Sipione S, Posse de Chaves E (2012). Beta-amyloid inhibits protein prenylation and induces cholesterol sequestration by impairing SREBP-2 cleavage. J Neurosci.

[92] Moreau K, Ravikumar B, Puri C, Rubinsztein DC (2012). Arf6 promotes autophagosome formation via effects on phosphatidylinositol 4,5-bisphosphate and phospholipase D. J Cell Biol.

[93] Morel E, Chamoun Z, Lasiecka ZM, Chan RB, Williamson RL, Vetanovetz C, Dall’Armi C, Simoes S, Point Du, Jour KS, McCabe BD, Small SA, Di Paolo G (2013). Phosphatidylinositol-3-phosphate regulates sorting and processing of amyloid precursor protein through the endosomal system. Nat Commun.

[94] Mucke L, Selkoe DJ (2012). Neurotoxicity of amyloid beta-protein: synaptic and network dysfunction. Cold Spring Harb Perspect Med.

[95] Mufson EJ, Ward S, Binder L (2014). Prefibrillar tau oligomers in mild cognitive impairment and Alzheimer’s disease. Neurodegener Dis.

[96] Neely Kayala KM, Dickinson GD, Minassian A, Walls KC, Green KN, Laferla FM (2012). Presenilin-null cells have altered two-pore calcium channel expression and lysosomal calcium: implications for lysosomal function. Brain Res.

[97] Neely KM, Green KN, LaFerla FM (2011). Presenilin is necessary for efficient proteolysis through the autophagy-lysosome system in a gamma-secretase-independent manner. J Neurosci.

[98] Nelson O, Tu H, Lei T, Bentahir M, de Strooper B, Bezprozvanny I (2007). Familial Alzheimer disease-linked mutations specifically disrupt Ca2+ leak function of presenilin 1. J Clin Invest.

[99] Nilsberth C, Westlind-Danielsson A, Eckman CB, Condron MM, Axelman K, Forsell C, Stenh C, Luthman J, Teplow DB, Younkin SG, Naslund J, Lannfelt L (2001). The ‘Arctic’ APP mutation (E693G) causes Alzheimer’s disease by enhanced Abeta protofibril formation. Nat Neurosci.

[100] Nishikawa T, Takahashi T, Nakamori M, Yamazaki Y, Kurashige T, Nagano Y, Nishida Y, Izumi Y, Matsumoto M (2014). Phosphatidylinositol-4,5-bisphosphate is enriched in granulovacuolar degeneration bodies and neurofibrillary tangles. Neuropathol Appl Neurobiol.

[101] Nixon RA, Wegiel J, Kumar A, Yu WH, Peterhoff C, Cataldo A, Cuervo AM (2005). Extensive involvement of autophagy in Alzheimer disease: an immuno-electron microscopy study. J Neuropathol Exp Neurol.

[102] Nixon RA, Yang DS (2011). Autophagy failure in Alzheimer’s disease—locating the primary defect. Neurobiol Dis.

[103] Noda T, Matsunaga K, Taguchi-Atarashi N, Yoshimori T (2010). Regulation of membrane biogenesis in autophagy via PI3P dynamics. Semin Cell Dev Biol.

[104] Obara K, Sekito T, Ohsumi Y (2006). Assortment of phosphatidylinositol 3-kinase complexes—Atg14p directs association of complex I to the pre-autophagosomal structure in Saccharomyces cerevisiae. Mol Biol Cell.

[105] Okamoto K, Hirai S, Iizuka T, Yanagisawa T, Watanabe M (1991). Reexamination of granulovacuolar degeneration. Acta Neuropathol.

[106] Panaretou C, Domin J, Cockcroft S, Waterfield MD (1997). Characterization of p150, an adaptor protein for the human phosphatidylinositol (PtdIns) 3-kinase. Substrate presentation by phosphatidylinositol transfer protein to the p150.Ptdins 3-kinase complex. J Biol Chem.

[107] Pickford F, Masliah E, Britschgi M, Lucin K, Narasimhan R, Jaeger PA, Small S, Spencer B, Rockenstein E, Levine B, Wyss-Coray T (2008). The autophagy-related protein beclin 1 shows reduced expression in early Alzheimer disease and regulates amyloid beta accumulation in mice. J Clin Invest.

[108] Platta HW, Stenmark H (2011). Endocytosis and signaling. Curr Opin Cell Biol.

[109] Polito VA, Li H, Martini-Stoica H, Wang B, Yang L, Xu Y, Swartzlander DB, Palmieri M, di Ronza A, Lee VM, Sardiello M, Ballabio A, Zheng H (2014). Selective clearance of aberrant tau proteins and rescue of neurotoxicity by transcription factor EB. EMBO Mol Med.

[110] Puri C, Renna M, Bento CF, Moreau K, Rubinsztein DC (2013). Diverse autophagosome membrane sources coalesce in recycling endosomes. Cell.

[111] Raemaekers T, Peric A, Baatsen P, Sannerud R, Declerck I, Baert V, Michiels C, Annaert W (2012). ARF6-mediated endosomal transport of Telencephalin affects dendritic filopodia-to-spine maturation. EMBO J.

[112] Raiborg C, Schink KO, Stenmark H (2013). Class III phosphatidylinositol 3-kinase and its catalytic product PtdIns3P in regulation of endocytic membrane traffic. FEBS J.

[113] Rajendran L, Annaert W (2012). Membrane trafficking pathways in Alzheimer’s disease. Traffic.

[114] Razi M, Chan EY, Tooze SA (2009). Early endosomes and endosomal coatomer are required for autophagy. J Cell Biol.

[115] Refolo LM, Eckman C, Prada CM, Yager D, Sambamurti K, Mehta N, Hardy J, Younkin SG (1999). Antisense-induced reduction of presenilin 1 expression selectively increases the production of amyloid beta42 in transfected cells. J Neurochem.

[116] Repetto E, Yoon IS, Zheng H, Kang DE (2007). Presenilin 1 regulates epidermal growth factor receptor turnover and signaling in the endosomal-lysosomal pathway. J Biol Chem.

[117] Roberson ED, Scearce-Levie K, Palop JJ, Yan F, Cheng IH, Wu T, Gerstein H, Yu GQ, Mucke L (2007). Reducing endogenous tau ameliorates amyloid beta-induced deficits in an Alzheimer’s disease mouse model. Science.

[118] Ruck A, Attonito J, Garces KT, Nunez L, Palmisano NJ, Rubel Z, Bai Z, Nguyen KC, Sun L, Grant BD, Hall DH, Melendez A (2011). The Atg6/Vps30/Beclin 1 ortholog BEC-1 mediates endocytic retrograde transport in addition to autophagy in *C*. *elegans*. Autophagy.

[119] Runz H, Rietdorf J, Tomic I, de Bernard M, Beyreuther K, Pepperkok R, Hartmann T (2002). Inhibition of intracellular cholesterol transport alters presenilin localization and amyloid precursor protein processing in neuronal cells. J Neurosci.

[120] Russell RC, Tian Y, Yuan H, Park HW, Chang YY, Kim J, Kim H, Neufeld TP, Dillin A, Guan KL (2013). ULK1 induces autophagy by phosphorylating Beclin-1 and activating VPS34 lipid kinase. Nat Cell Biol.

[121] Rusten TE, Stenmark H (2009). How do ESCRT proteins control autophagy?. J Cell Sci.

[122] Sahlin C, Lord A, Magnusson K, Englund H, Almeida CG, Greengard P, Nyberg F, Gouras GK, Lannfelt L, Nilsson LN (2007). The Arctic Alzheimer mutation favors intracellular amyloid-beta production by making amyloid precursor protein less available to alpha-secretase. J Neurochem.

[123] Saito T, Suemoto T, Brouwers N, Sleegers K, Funamoto S, Mihira N, Matsuba Y, Yamada K, Nilsson P, Takano J, Nishimura M, Iwata N, Van Broeckhoven C, Ihara Y, Saido TC (2011). Potent amyloidogenicity and pathogenicity of Abeta43. Nat Neurosci.

[124] Saitoh T, Fujita N, Jang MH, Uematsu S, Yang BG, Satoh T, Omori H, Noda T, Yamamoto N, Komatsu M, Tanaka K, Kawai T, Tsujimura T, Takeuchi O, Yoshimori T, Akira S (2008). Loss of the autophagy protein Atg16L1 enhances endotoxin-induced IL-1beta production. Nature.

[125] Sannerud R, Annaert W (2009). Trafficking, a key player in regulated intramembrane proteolysis. Semin Cell Dev Biol.

[126] Sannerud R, Declerck I, Peric A, Raemaekers T, Menendez G, Zhou L, Veerle B, Coen K, Munck S, De Strooper B, Schiavo G, Annaert W (2011). ADP ribosylation factor 6 (ARF6) controls amyloid precursor protein (APP) processing by mediating the endosomal sorting of BACE1. Proc Natl Acad Sci USA.

[127] Saura CA, Choi SY, Beglopoulos V, Malkani S, Zhang D, Shankaranarayana Rao BS, Chattarji S, Kelleher RJ, Kandel ER, Duff K, Kirkwood A, Shen J (2004). Loss of presenilin function causes impairments of memory and synaptic plasticity followed by age-dependent neurodegeneration. Neuron.

[128] Sawamura N, Gong JS, Garver WS, Heidenreich RA, Ninomiya H, Ohno K, Yanagisawa K, Michikawa M (2001). Site-specific phosphorylation of tau accompanied by activation of mitogen-activated protein kinase (MAPK) in brains of Niemann-Pick type C mice. J Biol Chem.

[129] Schu PV, Takegawa K, Fry MJ, Stack JH, Waterfield MD, Emr SD (1993). Phosphatidylinositol 3-kinase encoded by yeast VPS34 gene essential for protein sorting. Science.

[130] Schweitzer JK, Pietrini SD, D’Souza-Schorey C (2009). ARF6-mediated endosome recycling reverses lipid accumulation defects in Niemann-Pick Type C disease. PLoS One.

[131] Seaman MN (2012). The retromer complex - endosomal protein recycling and beyond. J Cell Sci.

[132] Settembre C, Di Malta C, Polito VA, Garcia Arencibia M, Vetrini F, Erdin S, Erdin SU, Huynh T, Medina D, Colella P, Sardiello M, Rubinsztein DC, Ballabio A (2011). TFEB links autophagy to lysosomal biogenesis. Science.

[133] Seubert P, Vigo-Pelfrey C, Esch F, Lee M, Dovey H, Davis D, Sinha S, Schlossmacher M, Whaley J, Swindlehurst C (1992). Isolation and quantification of soluble Alzheimer’s beta-peptide from biological fluids. Nature.

[134] Shravage BV, Hill JH, Powers CM, Wu L, Baehrecke EH (2013). Atg6 is required for multiple vesicle trafficking pathways and hematopoiesis in Drosophila. Development.

[135] Small SA, Kent K, Pierce A, Leung C, Kang MS, Okada H, Honig L, Vonsattel JP, Kim TW (2005). Model-guided microarray implicates the retromer complex in Alzheimer’s disease. Ann Neurol.

[136] Sou YS, Waguri S, Iwata J, Ueno T, Fujimura T, Hara T, Sawada N, Yamada A, Mizushima N, Uchiyama Y, Kominami E, Tanaka K, Komatsu M (2008). The Atg8 conjugation system is indispensable for proper development of autophagic isolation membranes in mice. Mol Biol Cell.

[137] Spampanato C, Feeney E, Li L, Cardone M, Lim JA, Annunziata F, Zare H, Polishchuk R, Puertollano R, Parenti G, Ballabio A, Raben N (2013). Transcription factor EB (TFEB) is a new therapeutic target for Pompe disease. EMBO Mol Med.

[138] Spasic D, Annaert W (2008). Building gamma-secretase: the bits and pieces. J Cell Sci.

[139] Spasic D, Tolia A, Dillen K, Baert V, De Strooper B, Vrijens S, Annaert W (2006). Presenilin-1 maintains a nine-transmembrane topology throughout the secretory pathway. J Biol Chem.

[140] Sun Q, Fan W, Chen K, Ding X, Chen S, Zhong Q (2008). Identification of Barkor as a mammalian autophagy-specific factor for Beclin 1 and class III phosphatidylinositol 3-kinase. Proc Natl Acad Sci USA.

[141] Sun Q, Westphal W, Wong KN, Tan I, Zhong Q (2010). Rubicon controls endosome maturation as a Rab7 effector. Proc Natl Acad Sci USA.

[142] Sun Q, Zhang J, Fan W, Wong KN, Ding X, Chen S, Zhong Q (2011). The RUN domain of rubicon is important for hVps34 binding, lipid kinase inhibition, and autophagy suppression. J Biol Chem.

[143] Szatmari Z, Kis V, Lippai M, Hegedus K, Farago T, Lorincz P, Tanaka T, Juhasz G, Sass M (2014). Rab11 facilitates cross-talk between autophagy and endosomal pathway through regulation of Hook localization. Mol Biol Cell.

[144] Takahashi RH, Almeida CG, Kearney PF, Yu F, Lin MT, Milner TA, Gouras GK (2004). Oligomerization of Alzheimer’s beta-amyloid within processes and synapses of cultured neurons and brain. J Neurosci.

[145] Thal DR, Del Tredici K, Ludolph AC, Hoozemans JJ, Rozemuller AJ, Braak H, Knippschild U (2011). Stages of granulovacuolar degeneration: their relation to Alzheimer’s disease and chronic stress response. Acta Neuropathol.

[146] Theuns J, Remacle J, Killick R, Corsmit E, Vennekens K, Huylebroeck D, Cruts M, Van Broeckhoven C (2003). Alzheimer-associated C allele of the promoter polymorphism −22C > T causes a critical neuron-specific decrease of presenilin 1 expression. Hum Mol Genet.

[147] Thimiri Govinda Raj DB, Ghesquiere B, Tharkeshwar AK, Coen K, Derua R, Vanderschaeghe D, Rysman E, Bagadi M, Baatsen P, De Strooper B, Waelkens E, Borghs G, Callewaert N, Swinnen J, Gevaert K, Annaert W (2011). A novel strategy for the comprehensive analysis of the biomolecular composition of isolated plasma membranes. Mol Syst Biol.

[148] Thoresen SB, Pedersen NM, Liestol K, Stenmark H (2010). A phosphatidylinositol 3-kinase class III sub-complex containing VPS15, VPS34, Beclin 1, UVRAG and BIF-1 regulates cytokinesis and degradative endocytic traffic. Exp Cell Res.

[149] Tokutake T, Kasuga K, Yajima R, Sekine Y, Tezuka T, Nishizawa M, Ikeuchi T (2012). Hyperphosphorylation of Tau induced by naturally secreted amyloid-beta at nanomolar concentrations is modulated by insulin-dependent Akt-GSK3beta signaling pathway. J Biol Chem.

[150] Tomiyama T, Matsuyama S, Iso H, Umeda T, Takuma H, Ohnishi K, Ishibashi K, Teraoka R, Sakama N, Yamashita T, Nishitsuji K, Ito K, Shimada H, Lambert MP, Klein WL, Mori H (2010). A mouse model of amyloid beta oligomers: their contribution to synaptic alteration, abnormal tau phosphorylation, glial activation, and neuronal loss in vivo. J Neurosci.

[151] Treusch S, Hamamichi S, Goodman JL, Matlack KE, Chung CY, Baru V, Shulman JM, Parrado A, Bevis BJ, Valastyan JS, Han H, Lindhagen-Persson M, Reiman EM, Evans DA, Bennett DA, Olofsson A, DeJager PL, Tanzi RE, Caldwell KA, Caldwell GA, Lindquist S (2011). Functional links between Abeta toxicity, endocytic trafficking, and Alzheimer’s disease risk factors in yeast. Science.

[152] Tsunemi T, Ashe TD, Morrison BE, Soriano KR, Au J, Roque RA, Lazarowski ER, Damian VA, Masliah E, La Spada AR (2012). PGC-1alpha rescues Huntington’s disease proteotoxicity by preventing oxidative stress and promoting TFEB function. Sci Transl Med.

[153] Tu H, Nelson O, Bezprozvanny A, Wang Z, Lee SF, Hao YH, Serneels L, De Strooper B, Yu G, Bezprozvanny I (2006). Presenilins form ER Ca2+ leak channels, a function disrupted by familial Alzheimer’s disease-linked mutations. Cell.

[154] Udayar V, Buggia-Prevot V, Guerreiro RL, Siegel G, Rambabu N, Soohoo AL, Ponnusamy M, Siegenthaler B, Bali J, Simons M, Ries J, Puthenveedu MA, Hardy J, Thinakaran G, Rajendran L (2013). A paired RNAi and RabGAP overexpression screen identifies Rab11 as a regulator of beta-amyloid production. Cell Rep.

[155] Umeda T, Tomiyama T, Sakama N, Tanaka S, Lambert MP, Klein WL, Mori H (2011). Intraneuronal amyloid beta oligomers cause cell death via endoplasmic reticulum stress, endosomal/lysosomal leakage, and mitochondrial dysfunction in vivo. J Neurosci Res.

[156] Vanier MT, Millat G (2003). Niemann-Pick disease type C. Clin Genet.

[157] Vellai T, Takacs-Vellai K, Sass M, Klionsky DJ (2009). The regulation of aging: does autophagy underlie longevity?. Trends Cell Biol.

[158] Volinia S, Dhand R, Vanhaesebroeck B, MacDougall LK, Stein R, Zvelebil MJ, Domin J, Panaretou C, Waterfield MD (1995). A human phosphatidylinositol 3-kinase complex related to the yeast Vps34p-Vps15p protein sorting system. EMBO J.

[159] Wahlster L, Arimon M, Nasser-Ghodsi N, Post KL, Serrano-Pozo A, Uemura K, Berezovska O (2013). Presenilin-1 adopts pathogenic conformation in normal aging and in sporadic Alzheimer’s disease. Acta Neuropathol.

[160] Walsh DM, Klyubin I, Fadeeva JV, Cullen WK, Anwyl R, Wolfe MS, Rowan MJ, Selkoe DJ (2002). Naturally secreted oligomers of amyloid beta protein potently inhibit hippocampal long-term potentiation in vivo. Nature.

[161] Wang JZ, Liu F (2008). Microtubule-associated protein tau in development, degeneration and protection of neurons. Prog Neurobiol.

[162] Wen L, Tang FL, Hong Y, Luo SW, Wang CL, He W, Shen C, Jung JU, Xiong F, Lee DH, Zhang QG, Brann D, Kim TW, Yan R, Mei L, Xiong WC (2011). VPS35 haploinsufficiency increases Alzheimer’s disease neuropathology. J Cell Biol.

[163] Willnow TE, Andersen OM (2013). Sorting receptor SORLA–a trafficking path to avoid Alzheimer disease. J Cell Sci.

[164] Wilson CA, Murphy DD, Giasson BI, Zhang B, Trojanowski JQ, Lee VM (2004). Degradative organelles containing mislocalized alpha-and beta-synuclein proliferate in presenilin-1 null neurons. J Cell Biol.

[165] Wolfe MS (2009). Tau mutations in neurodegenerative diseases. J Biol Chem.

[166] Wood DR, Nye JS, Lamb NJ, Fernandez A, Kitzmann M (2005). Intracellular retention of caveolin 1 in presenilin-deficient cells. J Biol Chem.

[167] Xiong H, Callaghan D, Jones A, Walker DG, Lue LF, Beach TG, Sue LI, Woulfe J, Xu H, Stanimirovic DB, Zhang W (2008). Cholesterol retention in Alzheimer’s brain is responsible for high beta- and gamma-secretase activities and Abeta production. Neurobiol Dis.

[168] Yamazaki Y, Matsubara T, Takahashi T, Kurashige T, Dohi E, Hiji M, Nagano Y, Yamawaki T, Matsumoto M (2011). Granulovacuolar degenerations appear in relation to hippocampal phosphorylated tau accumulation in various neurodegenerative disorders. PLoS One.

[169] Yang AJ, Chandswangbhuvana D, Margol L, Glabe CG (1998). Loss of endosomal/lysosomal membrane impermeability is an early event in amyloid Abeta1-42 pathogenesis. J Neurosci Res.

[170] Yang DS, Stavrides P, Mohan PS, Kaushik S, Kumar A, Ohno M, Schmidt SD, Wesson D, Bandyopadhyay U, Jiang Y, Pawlik M, Peterhoff CM, Yang AJ, Wilson DA, St George-Hyslop P, Westaway D, Mathews PM, Levy E, Cuervo AM, Nixon RA (2011). Reversal of autophagy dysfunction in the TgCRND8 mouse model of Alzheimer’s disease ameliorates amyloid pathologies and memory deficits. Brain.

[171] Yao J, Ho D, Calingasan NY, Pipalia NH, Lin MT, Beal MF (2012). Neuroprotection by cyclodextrin in cell and mouse models of Alzheimer disease. J Exp Med.

[172] Yu WH, Cuervo AM, Kumar A, Peterhoff CM, Schmidt SD, Lee JH, Mohan PS, Mercken M, Farmery MR, Tjernberg LO, Jiang Y, Duff K, Uchiyama Y, Naslund J, Mathews PM, Cataldo AM, Nixon RA (2005). Macroautophagy–a novel Beta-amyloid peptide-generating pathway activated in Alzheimer’s disease. J Cell Biol.

[173] Yue Z, Jin S, Yang C, Levine AJ, Heintz N (2003). Beclin 1, an autophagy gene essential for early embryonic development, is a haploinsufficient tumor suppressor. Proc Natl Acad Sci USA.

[174] Zeng X, Overmeyer JH, Maltese WA (2006). Functional specificity of the mammalian Beclin-Vps34 PI 3-kinase complex in macroautophagy versus endocytosis and lysosomal enzyme trafficking. J Cell Sci.

[175] Zhang X, Garbett K, Veeraraghavalu K, Wilburn B, Gilmore R, Mirnics K, Sisodia SS (2012). A role for presenilins in autophagy revisited: normal acidification of lysosomes in cells lacking PSEN1 and PSEN2. J Neurosci.

[176] Zhou X, Takatoh J, Wang F (2011). The mammalian class 3 PI3K (PIK3C3) is required for early embryogenesis and cell proliferation. PLoS One.

[177] Zhou X, Wang L, Hasegawa H, Amin P, Han BX, Kaneko S, He Y, Wang F (2010). Deletion of PIK3C3/Vps34 in sensory neurons causes rapid neurodegeneration by disrupting the endosomal but not the autophagic pathway. Proc Natl Acad Sci USA.

